# Engineering T cell receptor fusion proteins using nonviral CRISPR/Cas9 genome editing for cancer immunotherapy

**DOI:** 10.1002/btm2.10571

**Published:** 2023-07-10

**Authors:** Runzhe Shu, Maree Hammett, Vera Evtimov, Aleta Pupovac, Nhu‐Y Nguyen, Rasa Islam, Junli Zhuang, Seyeong Lee, Tae‐hun Kang, Kyujun Lee, Ian Nisbet, Peter Hudson, Jae Young Lee, Richard Boyd, Alan Trounson

**Affiliations:** ^1^ Cartherics Pty Ltd. Notting Hill Australia; ^2^ Australian Regenerative Medicine Institute Monash University Clayton Australia; ^3^ ToolGen Inc. Seoul South Korea; ^4^ Department of Obstetrics and Gynaecology Monash University Clayton Australia

**Keywords:** CAR‐T, CRISPR/Cas9, fusion protein, immunotherapy, nonviral

## Abstract

Manufacture of chimeric antigen receptor (CAR)‐T cells usually involves the use of viral delivery systems to achieve high transgene expression. However, it can be costly and may result in random integration of the CAR into the genome, creating several disadvantages including variation in transgene expression, functional gene silencing and potential oncogenic transformation. Here, we optimized the method of nonviral, CRISPR/Cas9 genome editing using large donor DNA delivery, knocked‐in an anti‐tumor single chain variable fragment (scFv) into the N‐terminus of CD3ε and efficiently generated fusion protein (FP) T cells. These cells displayed FP integration within the TCR/CD3 complex, lower variability in gene expression compared to CAR‐T cells and good cell expansion after transfection. CD3ε FP T cells were predominantly CD8^+^ effector memory T cells, and exhibited anti‐tumor activity in vitro and in vivo. Dual targeting FP T cells were also generated through the incorporation of scFvs into other CD3 subunits and CD28. Compared to viral‐based methods, this method serves as an alternative and versatile way of generating T cells with tumor‐targeting receptors for cancer immunotherapy.

## 1 INTRODUCTION

Chimeric antigen receptor (CAR)‐T cell therapy has achieved remarkable clinical success for patients with B cell malignancies, leading to the FDA approval of several autologous CAR‐T cell products.^1^, ^2^ Engineered by γ‐retroviral or lentiviral vectors, these products demonstrate applicability of viral delivery systems as clinical gene transfer tools.^3^ However, viral transduction results in random genome‐integration of the CAR gene, creating a number of disadvantages and risks that affect the quality of the resulting product.^4^, ^5^ Additionally, GMP‐grade viral vector manufacturing comprises a significant component of the total cost for CAR‐T cell therapies.^6^ Transposon‐based gene delivery systems serve as nonviral alternatives.^7^ However, these still carry comparable risks of random insertional mutagenesis and gene copy number variation, which can potentially lead to malignancy in patients.^8^, ^9^ Nonviral, precision integration methods for T cell gene engineering may overcome these challenges.

In the last decade, the CRISPR/Cas9 system has emerged as a cutting‐edge tool for simple, efficient and precise gene editing.^10^ Multiple FDA‐approved clinical trials utilizing CRISPR/Cas9 edited cell‐based therapies are underway for treating various genetic diseases including cancer.^11^, ^12^ Donor DNA, in conjunction with CRISPR/Cas9 editing, can target specific genome regions for more precise CAR gene integration via homology‐directed repair (HDR). Nonviral delivery of CRISPR/Cas9 components into T cells using electroporation achieves high editing efficiency and subsequent gene knockout (KO). However, it is challenging to deliver large DNA sequences, especially a CAR or T cell receptor (TCR) expression cassette, into T cells due to DNA toxicity.^13^

The endogenous TCR is a complex of six distinct receptor subunits that include the TCRα and TCRβ chains, and the CD3 signaling subunits which initiate T cell activation.^14^ In TCR‐based therapy, these components can be edited to direct specificity towards an antigen of interest; this approach shows promising outcomes.^15^ Bispecific antibodies engage the TCR with target tumor antigens, causing activation independent of the epitope specificity of the TCR.^16^ This principle motivated the engineering of single chain variable fragment (scFv)‐TCR/CD3 fusion protein (FP) T cells, which harness the signaling power of the endogenous TCR and endow direct anti‐tumor specificity through scFv engagement.

To engineer T cells without viral vectors, firstly we optimized our CRISPR/Cas9 method by testing multiple factors that can influence gene knock‐in (KI) efficiency. We found that reducing the length of the donor DNA is critical for KI efficiency and cell recovery. Considering the clinical approval of bispecific antibodies for cancer therapy, most of which activate T cells through engaging CD3ε in the TCR complex, we incorporated an scFv, rather than a CAR expression cassette, against two well‐known tumor antigen targets (CD19 or tumor‐associated glycoprotein 72; TAG‐72), into the N‐terminus of CD3ε to generate FP T cells. CD19, a B cell marker expressed highly and uniformly on leukemic B cells represents a “gold standard” target antigen in CAR‐T therapy.^2^ TAG‐72 is an oncofetal mucin that is overexpressed in most adenocarcinomas and some T cell lymphomas and is rarely present in normal post‐natal tissues.^17^, ^18^, ^19^ Targeting TAG‐72 using either antibody‐conjugates or CAR‐T cells has shown encouraging results.^20^, ^21^, ^22^ Using nonviral CRISPR/Cas9, TAG‐72 scFv or CD19 scFv were successfully incorporated within the endogenous TCR complex in human T cells. We showed that resultant CD3 FP T cells were capable of destroying cancer cells efficiently in vitro and in vivo. Moreover, we explored the possibility of improving CD3 FP T cell efficacy through dual targeting of TAG‐72 and CD19 as proof‐of‐concept.

## 2 RESULTS

### 2.1 Generation of TAG‐72/CD3ε FP T cells using nonviral CRISPR/Cas9

To establish the nonviral CRISPR/Cas9 gene KI method in human T cells, we tested the KI efficiencies of green fluorescent protein (GFP) or TAG‐72 CAR expression cassettes into the AAVS1 locus (Figure S1A).^23^ After demonstrating high guide RNA (gRNA) activity (Figure S1B), we observed that donor dsDNA showed a higher integration efficiency than plasmid donor DNA (Figure S1C). Nonviral delivery of the CAR cassette resulted in low KI efficiency and poor cell recovery (Figure S1D,E). Conversely, nonviral delivery of the GFP cassette demonstrated higher KI efficiency and better cell recovery compared to nonviral delivery of the CAR, which may be attributed to the shorter length of the cassette (Figure S1D,E). Additionally, we confirmed that reducing the length of the homology arms improved the nonviral KI efficiency (Figure S2). Overall, our data indicated that reducing donor dsDNA size improved KI efficiency and cell recovery after transfection.

**FIGURE 1:**
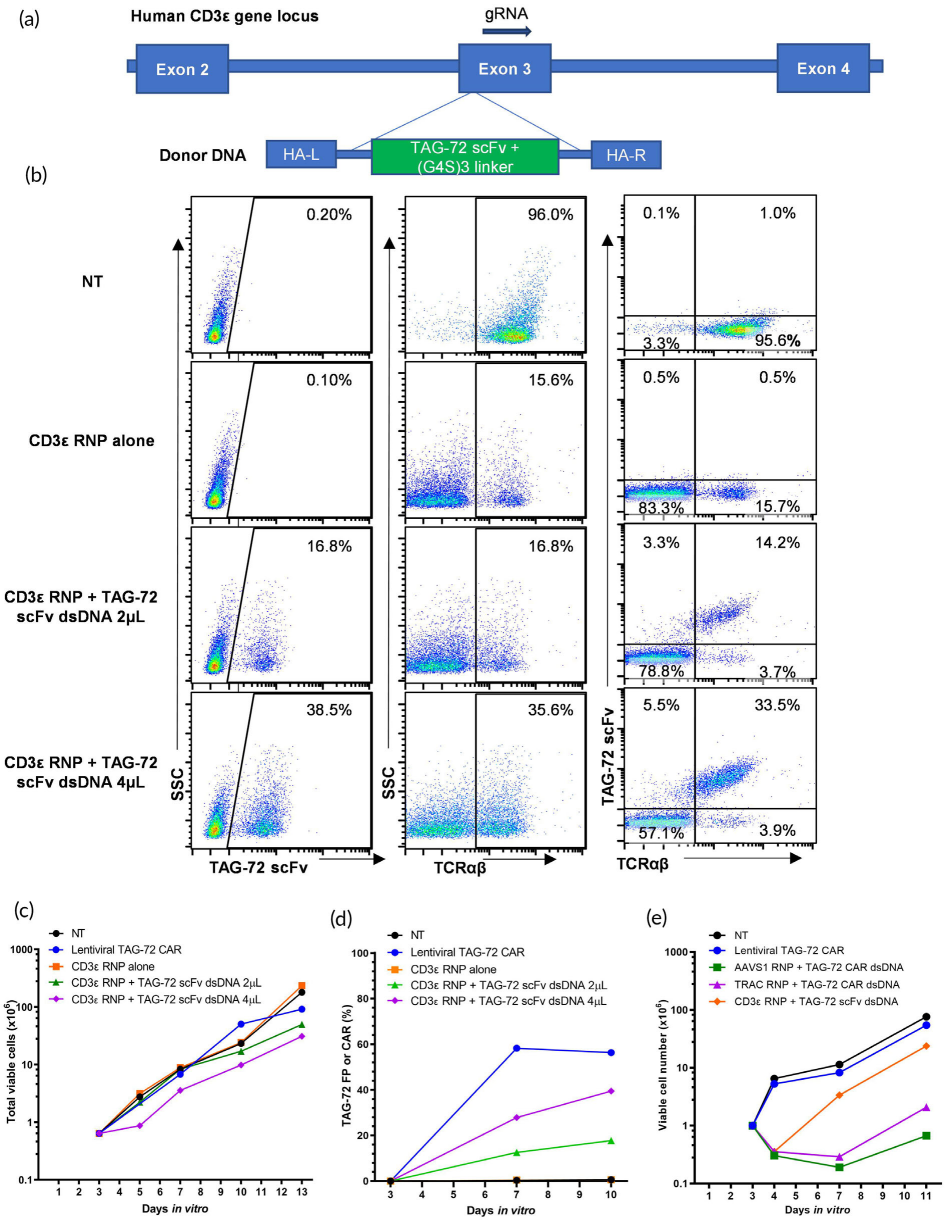
TAG‐72 scFv KI into the CD3ε locus generates TAG‐72/CD3ε FP T cells capable of expansion. (a) Schematic of a TAG‐72 scFv expression cassette for KI into the CD3ε locus. The donor DNA consists of two homologous arms (HA‐L and HA‐R), a TAG‐72 scFv and a (G4S)3 linker. Co‐transfection of CD3ε gRNA‐1 formed Cas9 RNP introduces the TAG‐72 scFv cassette into the CD3ε locus of activated human T cells. (b) Transfection efficiency (%) and surface expression of the TAG‐72 scFv and TCRαβ in nontransfected (NT) T cells, T cells transfected with CD3ε RNP alone, CD3ε RNP with 2 μL (low dose) or 4 μL (high dose) TAG‐72 scFv donor DNA (1.55 μg/μL) 10 days following activation. (c) Expansion of transfected TAG‐72 targeting T cells was tracked for 13 days following activation. Representative data are shown using T cells from one healthy donor. (d) TAG‐72 FP or CAR expression (%) is shown over 10 days of in vitro culture. Representative data are shown using T cells from one healthy donor. (e) Expansion of TAG‐72/CD3ε FP T cells was compared to CAR KI T cells. CAR KI T cells were created by knocking in the TAG‐72 CAR expression cassette into the AAVS1 or TRAC locus (3 μg dsDNA). NT T cells and lentiviral TAG‐72 CAR‐T cells were included as controls. Representative data are shown from two independent experiments (except for TRAC RNP + TAG‐72 CAR dsDNA data, which was from one independent experiment) using T cells from different healthy donors.

Our CD3 FP T cell KI strategy was to insert only the tumor binding moiety (scFv) from the CAR into the CD3 N‐terminus. The scFv is approximately 0.8 kb, which is considerably shorter than the entire CAR expression cassette (>3 kb) and therefore potentially amenable to nonviral KI into the T cell genome. Most bispecific T cell antibodies activate T cells through the binding of CD3ε.^16^ To investigate whether we could create FP T cells that mediate tumor killing using the same principle as bispecific antibodies, we knocked‐in a TAG‐72 scFv and a (G4S)3 linker into the N‐terminus of the CD3ε gene, linking the scFv with the CD3ε signaling subunits of the TCR complex to generate TAG‐72/CD3ε FP T cells (Figure 1a).

To utilize the endogenous signal peptide located at Exon 3 of the CD3ɛ gene, we designed two CRISPR gRNAs (CD3ε gRNA‐1 and CD3ε gRNA‐2) which introduce a double‐strand break after the CD3ɛ signal peptide. As a high gRNA activity is required for a good KI efficiency, CD3ε gRNA‐1 was selected based on its better KO efficiency compared to CD3ε gRNA‐2 (Figure S3A,B). Genome analysis also showed CD3ε gRNA‐1 introduced insertions and deletions (indels) into the CD3ε N‐terminus efficiently just after the endogenous CD3ε signal peptide sequence (Figure S3C,D). Next, we performed targeted deep sequencing of CD3ε gRNA‐1 on‐target and potential off‐target sites containing up to 3 bp mismatches in the human genome as identified by in silico analysis (Table S1). CD3ε gRNA‐1 showed high on‐target indel (92.3%) and one off‐target indel (<1.0%) throughout 20 off‐target sites tested (Figure S4).

To create the TAG‐72/CD3ε FP T cells, a low or high dose of TAG‐72 scFv donor dsDNA was knocked‐in to the CD3ɛ locus after co‐transfection with CD3ε gRNA‐1 RNP. By increasing the donor dsDNA dose, we achieved a 38.5% KI efficiency (Figure 1b). Transfection of CD3ε gRNA RNP alone reduced the TCRα/β positive T cells from 96.0% to 15.6% via CRISPR KO, while co‐transfection of CD3ε gRNA RNP and TAG‐72 scFv dsDNA rescued TCRα/β expression in a dose‐dependent manner (Figure 1b). This result indicated the TAG‐72/CD3ε FP was expressed on the cell surface after KI, and was successfully integrated into the TCR/CD3 complex. Alongside the TAG‐72/CD3ε FP T cells, we also generated TAG‐72 CAR‐T cells, which were previously shown to be effective in vitro and in vivo.^24^ Since lentiviral vectors are commonly used in CAR‐T cell engineering,^25^, ^26^ we compared the recovery and expansion of TAG‐72/CD3ε FP T cells to that of TAG‐72 CAR‐T cells. The dsDNA transfected at a higher dose resulted in more cell death after transfection, evidenced by a decrease in viable cell number after transfection (Day 5). However, both low or high dose TAG‐72 scFv dsDNA transfected cells could be recovered and expanded similarly to RNP control transfected cells, lentiviral TAG‐72 CAR and nontransfected (NT) T cells within 2 weeks (Figure 1c). TAG‐72/CD3ε FP or TAG‐72 CAR expression was tracked over 10 days of culture demonstrating stable transfection in the FP cells (Figure 1d). In contrast to TAG‐72/CD3ε FP T cells, TAG‐72 CAR nonviral KI T cells could not be expanded in 11 days (Figure 1e). These results indicated that TAG‐72/CD3ε FP T cells could be expanded in vitro within a timeframe comparable to viral transduced cells used for cell immunotherapy.

### 2.2 Characterization of TAG‐72/CD3ε FP T cells

**FIGURE 2:**
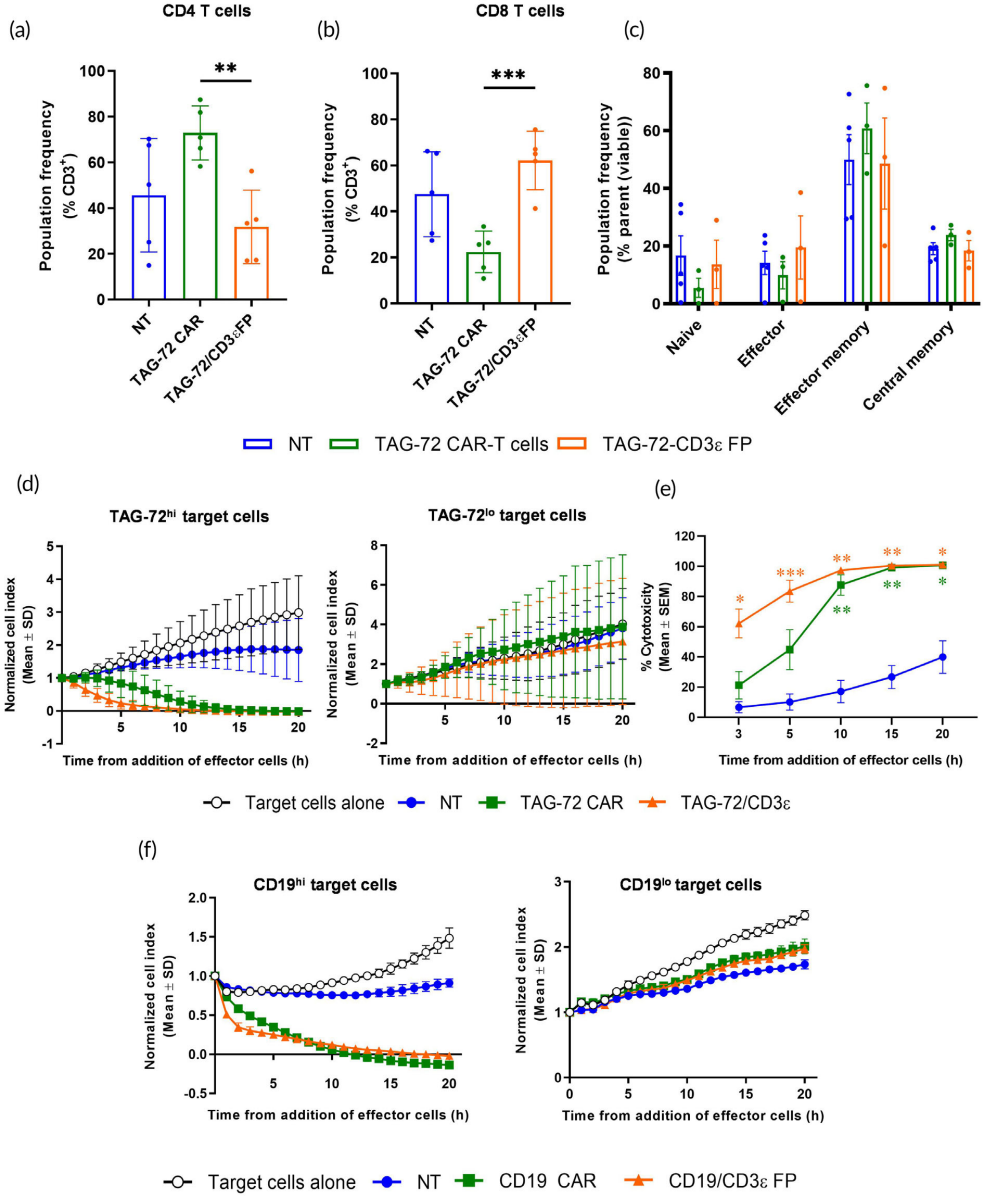
TAG‐72/CD3ε FP T cells are predominantly CD8^+^, effector memory T cells and both TAG‐72/CD3ε or CD19/CD3ε FP T cells mediate potent cell killing of respective tumor cell targets. (a) CD4, (b) CD8, (c) effector memory (CCR7^−^/CD45RO^+^), central memory (CCR7^+^/CD45RO^+^), naïve (CCR7^+^/CD45RO^−^) and effector cell (CCR7^−^/CD45RO^−^) frequencies were determined by flow cytometry. Data are represented as mean ± SD or *SEM* and are from 3 to 5 independent experiments using T cells from different healthy donors. **p ≤ 0.01, ***p ≤ 0.001 using a *t*‐test. (d) TAG‐72^hi^ OVCAR‐3 or TAG‐72^lo^ MES‐OV target cells were allowed to adhere to Real‐Time Cell Analyzer plates before addition of TAG‐72/CD3ε FP T cells, at an effector to target (E:T) ratio of 5:1. In parallel, nontransfected (NT) T cells and TAG‐72 CAR‐T cells were utilized as controls, and target cell proliferation under normal growth conditions was monitored. Cell impedance (represented as normalized cell index) was monitored over 20 h. (e) Percent cytotoxicity of TAG‐72/CD3ε FP T cells on TAG‐72^hi^ OVCAR‐3 target cells. (d,e) Data are represented as mean ± SD or *SEM* and are from four independent experiments (except in TAG‐72^lo^ MES‐OV experiments, which are from two independent experiments) using T cells from different healthy donors. *p ≤ 0.05, **p ≤ 0.01 using a two‐way analysis of variance (ANOVA). (f) CD19/CD3ε FP or CD19 CAR‐T cell induced cytotoxicity was assessed on CD19^hi^ or CD19^lo^ HeLa target cells by xCELLigence as described above. Data are shown using T cells from one healthy donor where intra‐assay duplicates or triplicates were used and are represented as mean ± SD.

To further characterize our FP T cells, we assessed the relative frequency of CD4^+^ and CD8^+^ cells, the prevalent T cell subset and functional ability of TAG‐72/CD3ε FP T cells compared to TAG‐72 CAR‐T cells. Less CD4^+^ but more CD8^+^ cells were present in TAG‐72/CD3ε FP T cells compared to TAG‐72 CAR‐T cells (Figure 2a,b). In addition, proportions of T cell subsets remained uniform, with effector memory (CCR7^−^/CD45RO^+^) the prevalent phenotype (Figure 2c). To evaluate the capacity of TAG‐72/CD3ε FP T cells to lyse tumor cells, high (OVCAR‐3) or low (ME‐SOV) TAG‐72 expressing cells were co‐cultured with TAG‐72/CD3ε FP T cells, TAG‐72 CAR‐T cells or negative control effector T cells. Cytotoxicity was determined by xCELLigence analysis. TAG‐72/CD3ε FP T cells killed TAG‐72^hi^ tumor cells as efficiently as TAG‐72 CAR‐T cells, whereas no lysis of TAG‐72^lo^ tumor cells was observed (Figure 2d). TAG‐72/CD3ε FP T cells also displayed faster killing of OVCAR‐3 cells compared to TAG‐72 CAR‐T cells (Figure 2e). To demonstrate that the method for generating TAG‐72/CD3ε FP T cells is not limited to only the TAG‐72 antigen, equivalent CD19/CD3ε FP T cells were generated, and the cytotoxicity of these cells was compared to CD19 CAR‐T cells. CD19/CD3ε FP T cells killed CD19^hi^ tumor cells (HeLa cells expressing CD19) as efficiently as the CD19 CAR‐T cells (Figure 2f).

### 2.3 Inclusion of an HDR enhancer or rho‐associated protein kinase (ROCK) inhibitor increases the KI efficiency and cell expansion of TAG‐72/CD3ε FP T cells

**FIGURE 3:**
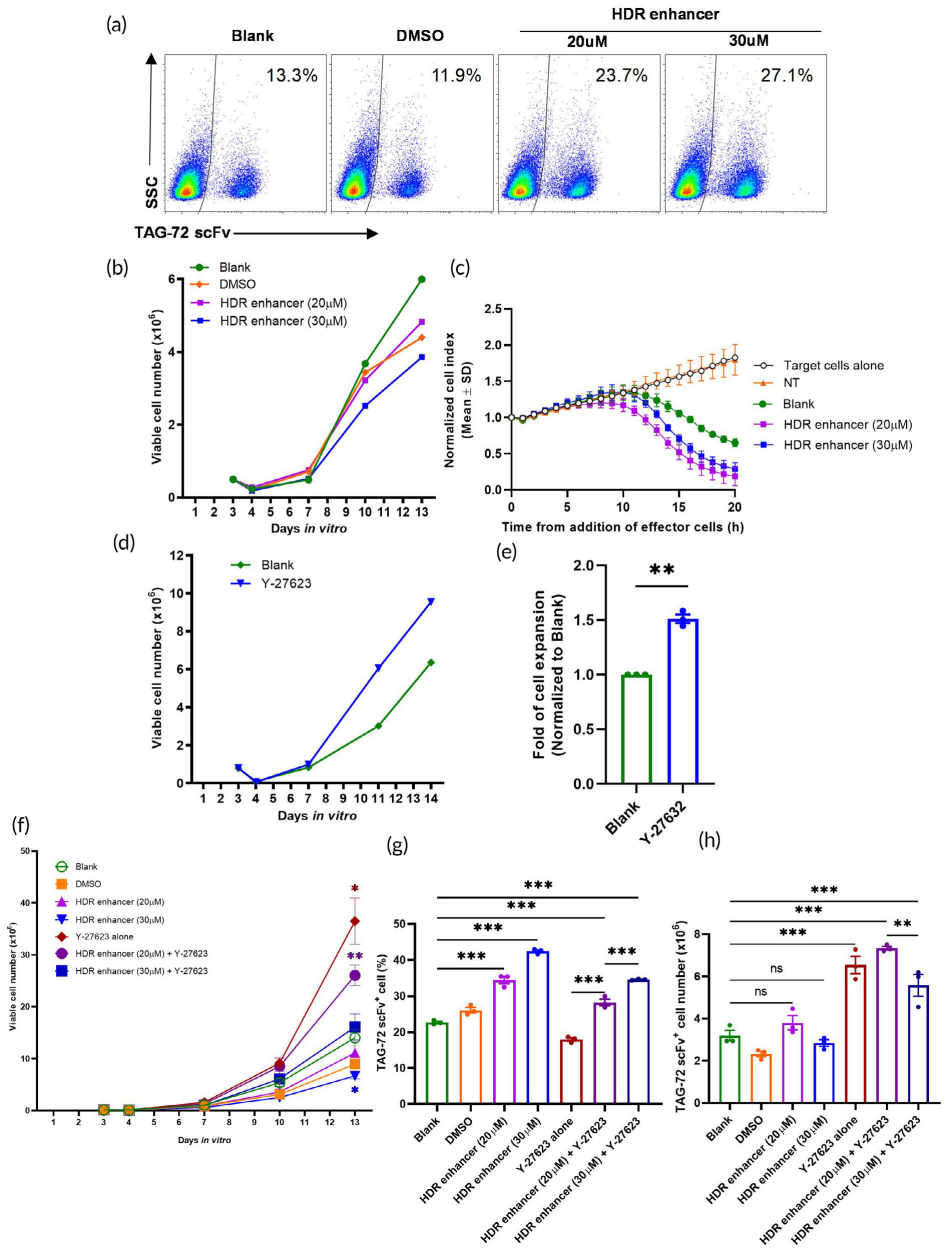
Addition of an HDR enhancer or ROCK inhibitor improves the yield of TAG‐72/CD3ε FP T cells in vitro. (a) Following nonviral CRISPR KI as described above, the transfection efficiency (%) of the TAG‐72 scFv after HDR enhancer or DMSO control treatment of cells was assessed by flow cytometry. Nontreated cells were included as a control (blank). (b) Expansion of TAG‐72/CD3ε FP T cells was measured over 10 days post transfection and treatment with the HDR enhancer. Representative data are shown using T cells from one healthy donor. (c) TAG‐72^hi^ OVCAR‐3 target cells were co‐cultured with TAG‐72/CD3ε FP T cells, and HDR enhancer treated TAG‐72/CD3ε FP T cells at an E:T ratio of 1:1 and cell impedance (normalized cell index) was monitored over 20 h. In parallel, nontransfected (NT) and untreated cells (blank), were utilized as controls and target cell proliferation under normal growth conditions was monitored. Representative data are shown using T cells from one healthy donor where intra‐assay duplicates or triplicates were used and are represented as mean ± SD. (d) Expansion of TAG‐72/CD3ε FP T cells was measured over 10 days post transfection in the presence or absence of Y‐27623. This is representative data of panel e and is shown using T cells from one healthy donor. (e) Fold expansion of TAG‐72/CD3ε FP T cells after 14 days of in vitro culture. Data are represented as mean ± *SEM* using T cells from three healthy donors. **p ≤ 0.01 by *t*‐test. (f) Expansion of TAG‐72/CD3ε FP T cells was measured over 10 days post transfection in the presence or absence of both the HDR enhancer and Y‐27623. (g) The percentage and (h) cell number of TAG‐72 scFv expressing cells were analyzed by flow cytometry at day 13 after expansion. (f–h) Data are represented as mean ± SD or *SEM* *p ≤ 0.05; **p ≤ 0.01; ***p ≤ 0.001 by one‐way or two‐way analysis of variance, ns, not significant. Three transfections were performed using T cells from one healthy donor.

CRISPR/Cas9 editing induces targeted DNA double‐strand breaks and activates subsequent repair pathways including the HDR pathway or the nonhomologous end‐joining (NHEJ) pathway. The HDR pathway operates at a lower efficiency than the NHEJ pathway, thus small molecule suppression of NHEJ can increase the efficiency of HDR and subsequent genetic modifications.^27^ Therefore, to further improve the KI efficiency when generating TAG‐72/CD3ε FP T cells, we assessed scFv KI efficiency and expansion following the addition of a commercially available HDR enhancer. An increase in KI efficiency was observed after the addition of the HDR enhancer at 20 and 30 μM (Figure 3a). However, compared to the DMSO vehicle control, the higher dose (30 μM) of the of HDR enhancer also inhibited cell expansion after transfection (Figure 3b) but the cytotoxicity of TAG‐72/CD3ε FP T cells after HDR enhancer treatment was not impaired (Figure 3c).

Y‐27632, is a ROCK inhibitor that is commonly used to improve cell viability during cryopreservation or electroporation of embryonic stem cells or induced pluripotent stem cells.^28^ Hence, we tested whether this inhibitor could improve cell viability and recovery following transfection. Y‐27632 improved TAG‐72/CD3ε FP T cell recovery (Figure 3d–f) and cell expansion after 2 weeks of in vitro culture (Figure 3e). Additionally, when cells were treated with Y‐27632 in the presence of an HDR enhancer, an increase in cell expansion (Figure 3f) percentage and number of TAG‐72‐positive cells (Figure 3g,h) was observed.

### 2.4 CD3ε FP T cells demonstrate anti‐tumor activity in vivo

**FIGURE 4:**
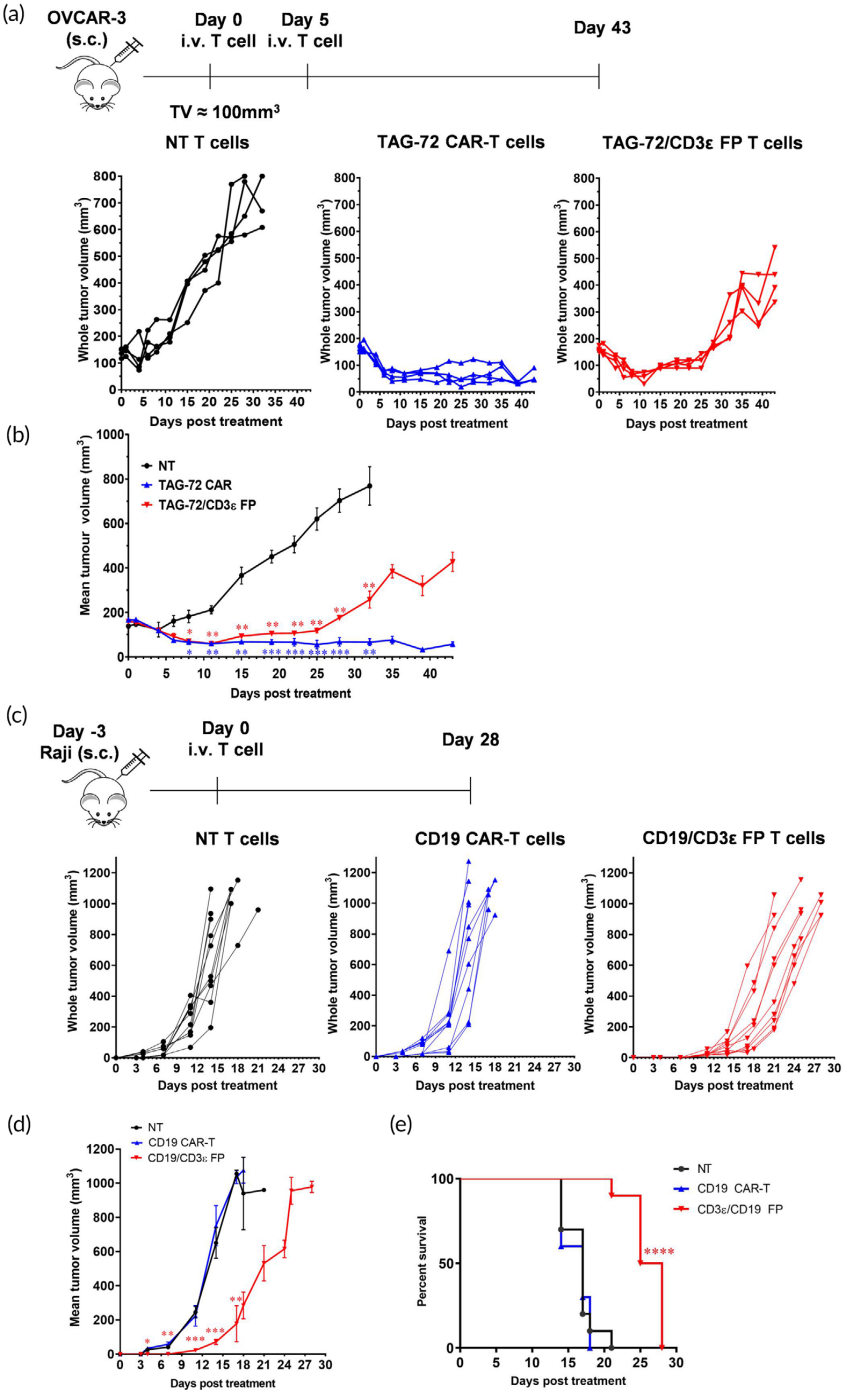
CD3ε FP T cells demonstrate anti‐tumor activity in vivo. (a,b) Schematic representation of the NSG OVCAR‐3 xenograft model. NSG mice bearing OVCAR‐3 derived tumors were treated at Day 0 (5 × 10^6^) and Day 5 (4 × 10^6^) with TAG‐72/CD3ε FP, TAG‐72 CAR‐T or nontransfected (NT) cells by intravenous injection (i.v.) when tumor volume (TV) reached approximately 100 mm^3^. TV was monitored for 43 days post first treatment. (b) Data represent mean ± *SEM* (*n* = 4, using T cells from one healthy donor), *p ≤ 0.05, **p ≤ 0.01, ***p ≤ 0.001 using a two‐way analysis of variance (ANOVA). (c,d) Schematic representation of the NSG Raji xenograft model. Three days after tumor inoculation, 5 × 10^6^ CD19/CD3ε FP, CD19 CAR‐T or NT cells were adoptively transferred by i.v. injection. Tumor volume was monitored until the termination of the experiment. (d) Data are represented as mean ± *SEM* (*n* = 10, from two independent experiments). *p ≤ 0.05, **p ≤ 0.01, ***p ≤ 0.001 using a two‐way ANOVA. (e) The percent survival of mice is indicated by the Kaplan–Meier curve and treatment groups were compared using the Log‐rank (Mantel–Cox) test and the Grehan–Breslow–Wilcoxon tests.

Having demonstrated the anti‐tumor activity of FP T cells in vitro, we subsequently assessed the potential efficacy of CD3ε FP T cells in vivo. An ovarian cancer cell xenograft mouse model was established by subcutaneous (s.c.) injection of TAG‐72^+^ OVCAR‐3 cells, and the tumor‐bearing NOD scid gamma (NSG) mice were allowed to establish a tumor mass of approximately 100 mm^3^ prior to injection with T cells. The anti‐tumor activity of subsequently administered TAG‐72/CD3ε FP T cells was compared to NT T cells and TAG‐72 CAR‐T cells. Both TAG‐72 CAR‐T cells and TAG‐72/CD3ε FP T cells displayed in vivo anti‐tumor activity as compared to the NT group, with TAG‐72 CAR‐T cells displaying a prolonged anti‐tumor response compared to TAG‐72/CD3ε FP T cells (Figure 4a,b). To observe whether this difference was donor‐related, we compared the activities of these three cell types generated from another donor for an additional 20 days (up to day 60). We observed similar tumor suppression up to 25 days, with TAG‐72 CAR‐T cells displaying a prolonged anti‐tumor response compared to TAG‐72/CD3ε FP T cells (Figure S5). Next, NSG mice were s.c. inoculated with the Raji cell line, a CD19^+^ Burkitt lymphoma cell line, to evaluate the anti‐tumor activity of CD19/CD3ε FP T cells and CD19 CAR‐T cells. CD19/CD3ε FP T cells were administrated into mice 3 days after tumor inoculation. In this aggressive and fast‐growing tumor model, CD19/CD3ε FP T cells delayed tumor growth (Figure 4c) and mice showed significant survival advantage compared to NT T cell and CD19 CAR‐T cell treated mice (Figure 4d,e). In summary, compared to NT T cells, CD3ε FP T cells demonstrated anti‐tumor activity in two different in vivo tumor models. However, the relative efficacy of CD3ε FP T cells compared to lentiviral CAR‐T cells needs to be further evaluated across different tumor models and tumor targets.

### 2.5 Generation and characterization of TAG‐72/CD3ε, δ or γ FP T cells

**FIGURE 5:**
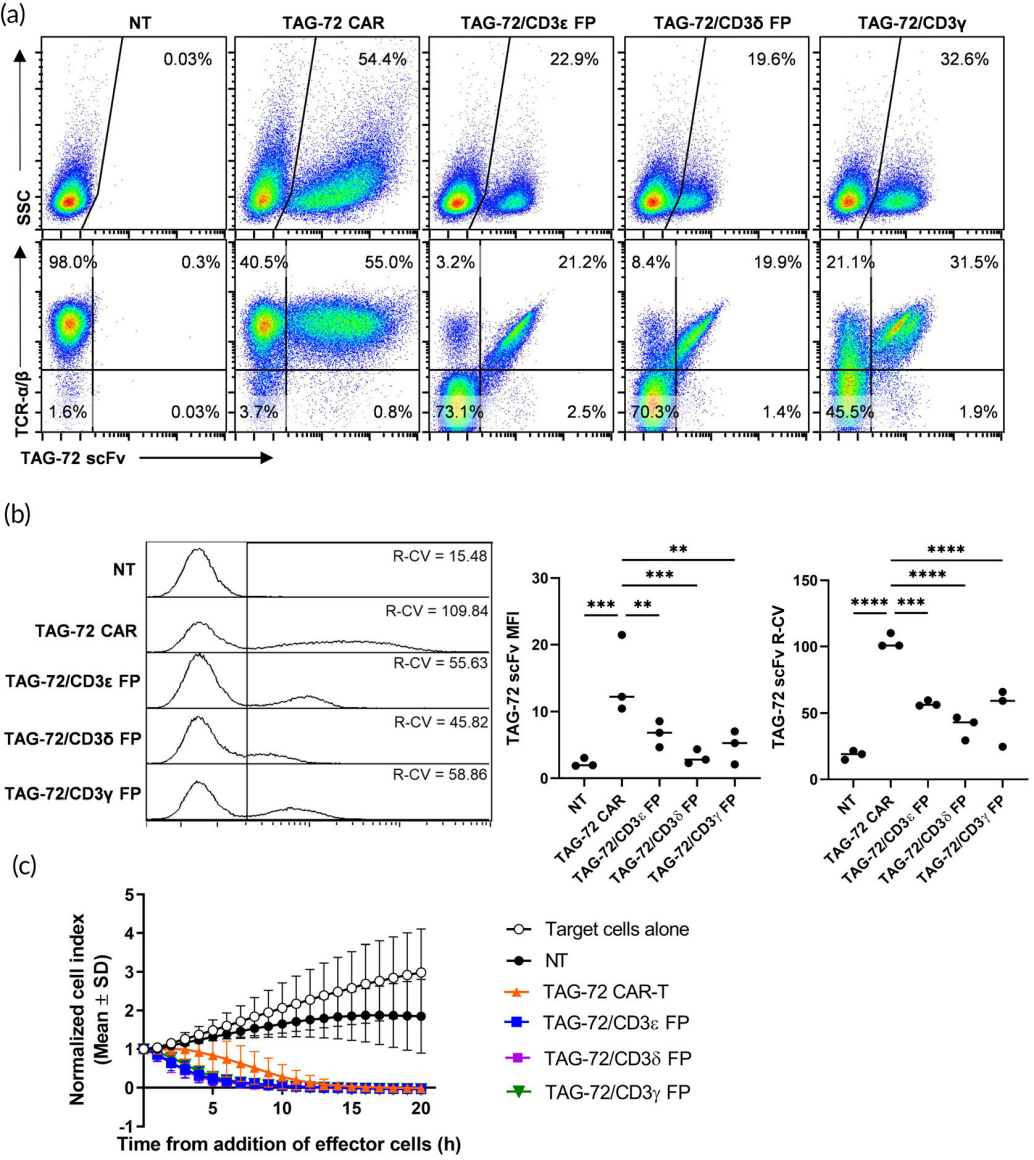
TAG‐72 scFv KI into the CD3ε, CD3δ or CD3γ N‐terminal loci generates functional TAG‐72/CD3ε, δ or γ FP T cells respectively. TAG‐72/CD3ε, δ or γ FP T cells were generated as described above and (a) the expression (%) of TAG‐72 scFv and TCRα/β was analyzed by flow cytometry 10 days after transfection. TAG‐72 CAR‐T cells were included for comparative purposes and nontransfected (NT) T cells as controls. (b) TAG‐72 scFv mean fluorescence intensity (MFI) and the MFI robust coefficient of variation (R‐CV) were determined for transduced and transfected cells. MFI was used to compare the surface expression level of the TAG‐72 scFv and R‐CV was used to measure the dispersion of scFv expression. Data are represented as median MFI and R‐CV. Cells were generated from three different healthy donors **p ≤ 0.01, ***p ≤ 0.001, ****p ≤ 0.0001 using a one‐way analysis of variance. (c) TAG‐72/CD3ε, δ or γ FP T cells mediate potent cell killing of TAG72^hi^ target cells in vitro. OVCAR‐3 target cells were co‐cultured with NT T cells, lentiviral TAG‐72 CAR‐T cells, or TAG‐72/CD3ε, δ or γ FP T cells at an E:T ratio of 5:1, and the cytotoxic response was monitored in real time using xCELLigence. Data are represented as mean ± SD and are from four independent experiments (except TAG‐72/CD3δ and TAG‐72/CD3γ FP data, which are from two independent experiments) using T cells from different healthy donors.

The CD3 signaling proteins associated with the TCRα and TCRβ heterodimer are comprised of one CD3ε/γ dimer, one CD3ε/δ dimer, and one CD3ζ homodimer.^29^, ^30^ To demonstrate that the method for generating TAG‐72 FP T cells is not limited to just the ε chain, equivalent TAG‐72 FP T cells were generated via CD3δ or CD3γ chain. TAG‐72 CAR‐T cells were included for comparative purposes. Lentiviral transduced CARs usually display heterogeneous expression in cells due to random integration of varied copy numbers of the transgene, which is driven by constitutive promoters such as EF1α.^31^ Lentiviral transduction of the TAG‐72 CAR yielded a high transduction efficiency, and the expression of the TAG‐72 scFv in the CAR was independent of TCRα/β expression. In contrast, TAG‐72 scFv expression in CD3ε, CD3δ and CD3γ FP cells correlated with TCRα/β expression, indicating the TAG‐72 CD3 FPs were incorporated with the TCR/CD3 complex on the cell surface (Figure 5a). Although TAG‐72/CD3ε, δ or γ FP T cells showed a lower mean expression (indicated by mean fluorescence intensity; MFI) than lentiviral CAR‐T cells, they displayed more homogenous and consistent transgene expression across multiple donors (Figure 5b). These results suggest that TAG‐72/CD3 FP expression is under the regulatory control of CD3 native cis‐regulatory elements in CD3 FP T cells. TAG72/CD3ε, δ and γ FP T cells were subsequently tested for their ability to kill tumor cells in vitro. All three types of FP T cells induced the in vitro killing of TAG‐72^hi^ tumor cells as effectively as the TAG‐72 CAR‐T cells (Figure 5c).

### 2.6 Generation of dual antigen targeting CD3 FP T cells

**FIGURE 6:**
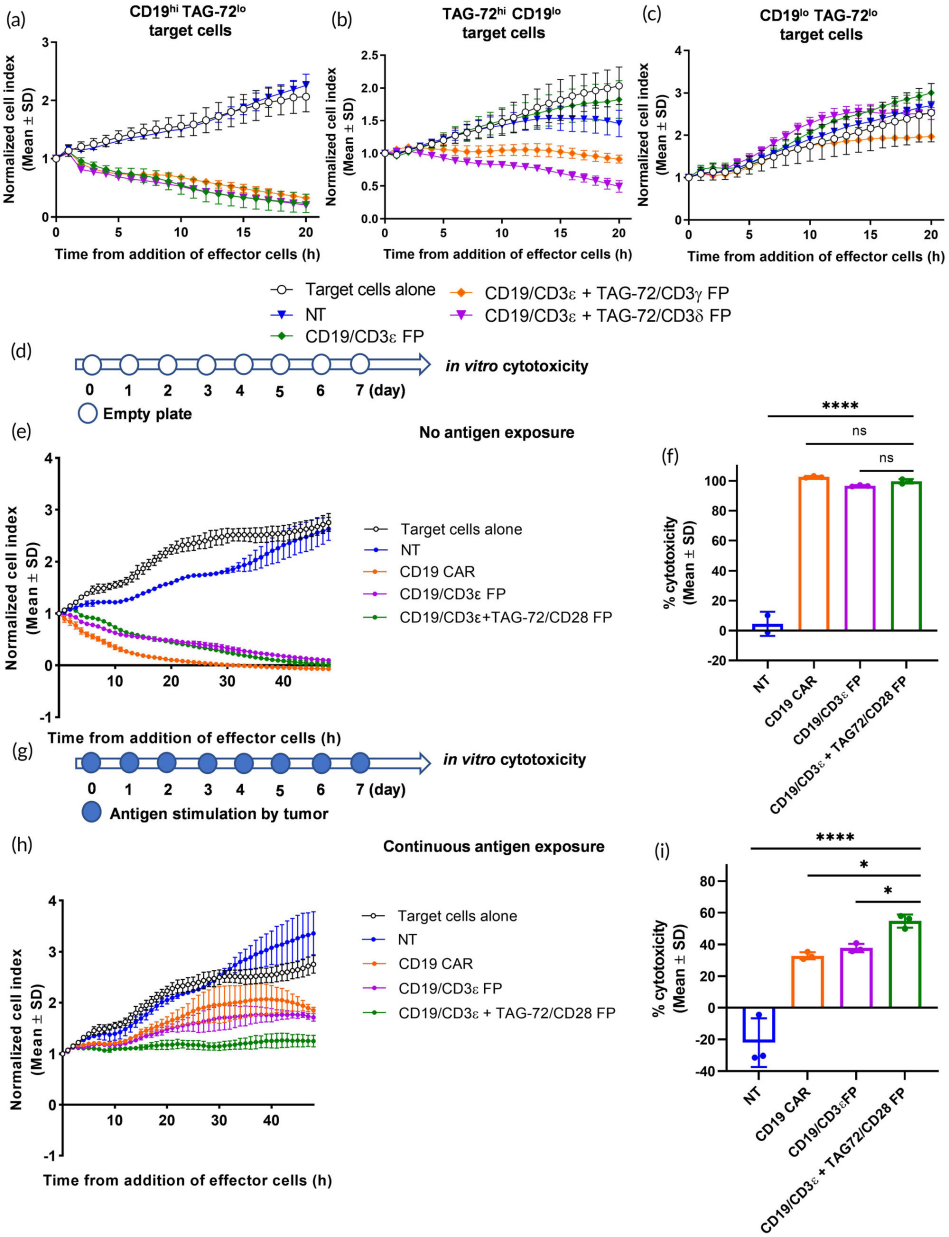
Dual antigen targeting FP T cells enhance target cell killing. (a) CD19^hi^ TAG‐72^lo^ target tumor cells (CD19 stable expressing HeLa), (b) TAG‐72^hi^ CD19^lo^ tumor cells (OVCAR‐3), or (c) CD19^lo^ TAG‐72^lo^ tumor cells (HeLa) were co‐cultured with CD19/CD3ε single targeting FP T cells, CD19/CD3ε + TAG‐72/CD3γ or CD19/CD3ε + TAG‐72/CD3δ dual antigen targeting FP T cells (E:T = 1:1). In parallel, target cells alone and nontransfected (NT) T cells were utilized as controls. Data are represented as mean ± SD (technical duplicates or triplicates), where CAR and FP T cells were generated using T cells from one healthy donor. The effect of continuous antigen exposure was assessed by incubating (d–f) CD19/CD3ε + TAG‐72/CD28 dual FP, CD19/CD3ε FP, CD19 CAR or NT T cells alone without antigen stimulation or with (g–i) CD19^hi^ TAG‐72^hi^ target tumor cells (CD19 overexpressing OVCAR‐3 cell line) for 7 days. (e,h) The cytotoxic response was monitored using xCELLigence and (f,i) the percentage of cytotoxicity (relative to target cells alone maintained under normal growth conditions) was determined following 48 h of co‐culture. Data represented as the mean ± SD using the T cells from a single healthy donor in technical duplicate or triplicate. *p < 0.05, ****p < 0.0001 by one‐way analysis of variance, ns is not significant.

Dual antigen targeting is a common strategy employed to avoid tumor escape and may improve the effectiveness of immunotherapies.^32^ Since CD3ε, δ and γ subunits of TCR can be engineered to create cytotoxic FP T cells, distinct scFv sequences could be fused with different CD3 subunits to create multiple antigen‐targeting CD3 FP T cells. To test this hypothesis, the CD19 scFv was knocked‐in to the CD3ε locus, while the TAG‐72 scFv was knocked‐in to either the CD3δ or γ loci. As expected, all three types of T cells with CD19/CD3ε FP expression killed CD19^hi^ TAG‐72^lo^ targets effectively compared to NT control T cells (Figure 6a). Moreover, the dual targeting CD19/CD3ε + TAG‐72/CD3δ or CD19/CD3ε + TAG‐72/CD3γ FP T cells killed TAG‐72^hi^ CD19^lo^ tumor cells, while the CD19/CD3ε single targeting FP T cells could not eradicate CD19^lo^ cells (Figure 6b). All cells were unable to kill CD19^lo^ TAG‐72^lo^ targets, indicating that dual targeting FP T cells were specific for their targets (Figure 6c). These data suggest that this method can be used to target multiple tumor antigens.

Costimulatory signaling, provided by molecules such as CD28 or 4‐1BB in traditional CAR constructs, play an important role in the effectiveness and safety of CAR‐T therapy.^33^ Primary TCR signaling was activated in CD19/CD3ε FP T cells, therefore, to include costimulatory signaling in FP T cells, we knocked‐in TAG‐72 into the CD28 locus and created TAG‐72/CD28 FP T cells. However, these cells could not mediate in vitro tumor killing alone (Figure S6). Through double KI, CD19/CD3ε + TAG‐72/CD28 FP T cells were created to trigger both TCR primary and costimulatory signaling. Freshly prepared CD19/CD3ε FP T, CD19/CD3ε + TAG‐72/CD28 FP T and CD19 CAR‐T cells were all able to eliminate CD19^hi^ TAG‐72^hi^ target cells efficiently in vitro (Figure 6d–f). After 7 days of continuous antigen exposure to CD19^hi^ TAG‐72^hi^ tumor cells, all groups of T cells were driven to an exhausted or dysfunctional state, evidenced by a reduced percent cytotoxicity (Figure 6g–i). However, CD19/CD3ε + TAG‐72/CD28 FP T cells remained more cytotoxic than CD19/CD3ε FP and CD19 CAR‐T cells after long term antigen exposure (Figure 6h,i). These data demonstrated that FP cells could be created through the redirection of both TCR/CD3 signaling and CD28 costimulatory signaling, and this dual activation strategy could be an approach to further enhance the persistence of CD3 FP T cells.

## 3 DISCUSSION

Despite the potential exhibited by T cell products generated using viral transduction, they still possess several limitations.^25^ We developed a nonviral gene editing technology using CRISPR/Cas9, to create cancer antigen (TAG‐72 or CD19) targeting FP T cells applicable to cancer immunotherapy. Following gene editing, we observed good cell recovery, expansion and function; cells were generated in a timeframe on par with that of CAR‐T cells. The method created precisely gene‐edited, antigen targeting T cells using a relatively simple and straightforward method, which is scalable for clinical application and may be useful as a tool for early discovery.

Multiple strategies can be used to deliver CRISPR/Cas9 protein or mRNA into cells without viral vectors, including lipid nanoparticles, cell‐penetrating peptides and extracellular vesicles.^34^ While these new strategies are promising, electroporation or nucleofection remains a gold standard in efficiently delivering large donor DNA via CRISPR/Cas9 into T cells.^35^, ^36^, ^37^ To improve the T cell nucleofection method, we tested multiple factors that can influence gene KI efficiency and subsequent expansion. In HEK293T cells, KI of dsDNA is more efficient than plasmid donor DNA, and editing efficiency also depends on dsDNA size.^38^ Our data confirm that using dsDNA achieves better T cell KI efficiency compared to traditional plasmid donor DNA. Following the shortening of homology arm length, we obtained a better GFP KI efficiency, as previously reported.^35^ However, the larger size of the CAR coding sequence, in comparison to the GFP coding sequence, required longer homology arms for efficient incorporation. This negatively impacted T cell recovery and expansion after transfection, which may be a major hurdle for CAR cassette insertion. Our CD3 FP T cell strategy utilizes the endogenous cis‐regulatory elements of CD3ε and does not require inclusion of a promoter, signal peptide and polyA terminator sequences for transgene expression. The scFv, being similar in size to GFP, but smaller than a CAR, allowed for a reduction of homology arm length and resulted in the subsequent improvement of KI efficiency and cell recovery.

While the nonviral CRISPR/Cas9 method utilized in this study is convenient and promising, it remains important to assess the safety of FP T cells at various levels before considering their clinical application. Off‐target genome editing poses a potential risk that should be considered. We examined the possibility of off‐target editing associated with CD3ε gRNA‐1, and identified one off‐target editing in the intron region of the *DOCK1* gene. Importantly, it should be noted that this gene is reported to have no expression in lymphocytes.^39^ Though the overall off‐target effects were low, the impacts on the genome integrity and T cell function need further study before considering clinical application.

Although the inclusion of costimulatory signaling is required for maximal clinical CAR‐T cell efficacy, it can be responsible for inducing adverse events such as cytokine release syndrome in patients.^33^ T cells with CAR expression driven by an endogenous TCR promoter, instead of constitutive promoters could be more persistent in vivo.^31^ Additionally, several reports have proposed that nonnatural activation results in over‐stimulation or tonic signaling of CAR‐T cells, leading to exhaustion and impaired anti‐tumor effects.^40^, ^41^ Thus, engineered TCR‐T cells are an alternative to CAR‐T cells for adoptive T cell therapy. We showed that TAG‐72/CD3ε FP T cells recovered and expanded following transfection. TAG‐72/CD3ε FP T cells were also comprised of a higher CD8^+^ cytotoxic population, which may contribute to their faster in vitro tumor cell killing.

Multiple chemical‐based approaches may be used to improve CRISPR/Cas9 gene editing efficiency.^42^ Our results indicated that the small molecule HDR enhancer tested here could improve the KI efficiency without impairing T cell cytotoxicity. Although this enhancer adversely affected T cell expansion, more small molecules associated with enhancement or inhibition of the HDR or NHEJ pathways could be assessed in the future.^43^, ^44^, ^45^ We also showed that the ROCK inhibitor Y‐27632, a compound usually used to improve stem cell viability post‐thawing and during cryopreservation, also improved our T cell recovery and expansion after transfection. The ROCK inhibitor alone significantly increased the final yield of FP T cells with or without HDR enhancer, indicating that reducing DNA‐induced toxicity and improving cell recovery must be first considered.

We used two tumor xenograft mouse models to evaluate the in vivo anti‐tumor activity of CD3ε FP T cells: a tumor model based on the ovarian cancer OVCAR‐3 cell line, and a tumor model based on the rapidly growing B cell lymphoma Raji cell line. All CD3ε FP T cells displayed anti‐tumor activity relevant to the specific tumor target, however TAG‐72/CD3 FP T cells could not suppress tumor growth for as long as the lentiviral transduced TAG‐72 CAR‐T cells in the established ovarian cancer cell tumor model. On the other hand, CD19/CD3ε FP T cells performed better than the lentiviral CD19 CAR‐T cells in the fast‐growing B cell tumor model. CD3ε FP T cells were comprised of more CD8^+^ T cells and less CD4^+^ T cells compared to lentivirus transduced CAR‐T cells. Most cell therapy products are a combination of CD8 and CD4 T cells, and in terms of anti‐tumor killing dynamics, CD4 CAR‐T cells are slower, less prone to exhaustion, and are more persistent following antigen exposure compared to CD8 T cells. But CD8 T cells are believed to be the main contributor to the anti‐tumor efficacy of cell therapy, and CD8 CAR‐T cell adoptive transfer alone has been shown to be sufficient for long‐term B cell eradication.^46^

Solid tumors are notoriously difficult to tackle due to T cell exhaustion following continuous antigen exposure.^47^ Our in vitro data indicated that dual antigen targeting could overcome antigen‐low escape; when dual antigen targeting utilized both CD3 and CD28 costimulatory signaling, the dual targeting FP T cells were more resistant to continuous antigen exposure induced exhaustion compared to single antigen targeting FP T cells. Future studies should evaluate the efficacy of dual antigen targeting FP T cells in in vivo tumor models to ascertain whether they reduce the rate of tumor relapse. Furthermore, two lentiviral engineered TCR‐T cell studies, antibody‐TCR^48^ and TCR fusion construct^49^ T cells, which engage T cells only through the TCR complex, also displayed faster and more efficient killing than CAR‐T cells in Raji cell xenograft models.

## 4 MATERIALS AND METHODS

### 4.1 Human T cell isolation and culture

Primary human T cells were isolated from healthy human donors obtained from Australian Red Cross Lifeblood. All healthy donors provided informed consent. Peripheral blood mononuclear cells (PBMCs) were isolated by Ficoll‐Paque (GE Healthcare, Chicago, IL, USA)‐mediated centrifugation using Leucosep® tubes (Greiner Bio‐One, Kremsmünster, Austria) as per the manufacturer's instructions. PBMCs were cryopreserved until use. Prior to transfection or lentiviral transduction, PBMCs were thawed and isolated using Dynabeads™ human T‐expander CD3/CD28 beads (Thermo Fisher Scientific, Waltham, MA, USA) and subsequently activated for 72 h at a bead to cell ratio of 3:1 in the presence of 100 IU/mL interleukin (IL)‐2 (Miltenyi Biotec, Bergisch Gladbach, Germany). Beads were removed following activation.

### 4.2 Lentiviral CAR‐T cell production

Lentiviral CAR vector transduced activated human T cells (referred to throughout as CAR‐T cells) were used as a positive control. CAR constructs were generated with an scFv for TAG‐72, which was validated in CAR format as previously described^50^, ^51^, ^52^ and a comparable approach was taken for CD19 using the FMC63 scFv. Following a conventional human secretion signal leader, the scFv was constructed in a variable heavy chain‐linker‐variable light chain orientation with a 15‐residue (G4S)3 linker. The CAR constructs used human CD8 as hinge and transmembrane regions, and 4‐1BB and CD3ζ cytoplasmic signaling domains. The second‐generation lentiviral packaging system was used to produce the lentiviral vectors for CAR transduction.^24^, ^53^ Following activation, bead‐free T cells were incubated with lentiviral particles in RetroNectin® (Takara Bio, Kusatsu, Japan)‐coated plates for 48 h at a multiplicity of infection of 50 in the presence of 200 U/mL IL‐2. Following removal of virus, T cell cultures were transferred to complete T cell expansion media comprising of IL‐2, IL‐7, IL‐15, IL‐21 (Miltenyi Biotec), human AB (hAb) serum (Sigma‐Aldrich, St. Louis, MO, USA) and Stemulate® (Cook Regentec, Indianapolis, IN, USA) in TexMACS™ (Miltenyi Biotec) for continued expansion.

### 4.3 gRNA and RNP formation

Cas9 RNPs were prepared as previously described.^50^, ^51^ In short, spCas9 protein (Integrated DNA Technologies, Coralville, IA, USA or Aldevron, Fargo, ND, USA) was incubated with chemical‐modified synthetic gRNAs (Synthego, Redwood City, CA, USA or Integrated DNA Technologies) at ratios ranging from 1:2 to 1:3 in P3 transfection buffer (Lonza, Basel, Switzerland) at room temperature for 15 min. The gRNAs used for FP T cell engineering were as follows: CD3ε gRNA‐1: GTTGGCGTTTGGGGGCAAGA; CD3ε gRNA‐2: ATTTTCTAGTTGGCGTTTGG; CD3δ gRNA: CCTCTATAGGTATCTTGAAG; CD3γ gRNA: ACTTTGGCCCAGTCAATCAA; CD28 gRNA: TCGTCAGGACAAAGATGCTC.

### 4.4 Donor DNA production

Donor DNA sequences for HDR were constructed by assembling gBlocks™ gene fragments (Integrated DNA Technologies) using NEBuilder® HiFi DNA Assembly Cloning Kit (New England Biolabs, Ipswich, MA, USA). Homology arms and the desired inserts of all constructs were introduced into a cloning vector as indicated in Figures S1 and 1. The AAVS1 or TRAC CAR or GFP donor DNA contained the human elongation factor‐1 alpha (EF‐1α) constitutive promoter, either the TAG‐72 CAR, CD19 CAR or GFP, and the Bgh‐PolyA sequence, flanked by two 800 bp homology arms. The CD3 or CD28 FP donor DNA contained either the TAG‐72 or CD19 scFv, a FLAG tag, a short hinge and (G4S)3 linker, flanked by two 300 bp homology arms. These plasmids were used as templates for dsDNA PCR amplification. dsDNA was amplified using Q5® High‐Fidelity Taq polymerase (New England Biolabs) or PrimerSTAR® MAX DNA polymerase (Takara Bio, Kusatsu, Japan) with primers and then purified by the QIAquick® PCR purification kit (QIAGEN, Hilden, Germany). Concentrations of dsDNA were determined by NanoDrop. The size of the amplified dsDNA was confirmed by agarose gel electrophoresis. All DNA and primer sequences were as previously described.^51^

### 4.5 T cell nucleofection

Nucleofection was performed using the 4D‐Nucleofector™ system (Lonza, Basel, Switzerland). For optimal editing, 0.5–3 × 10^6^ activated human T cells were washed by D‐PBS, then resuspended into 20 μL P3 transfection buffer (Lonza, Basel, Switzerland). 5 μL of Cas9 RNP, 40 pmols spCas9 and gRNAs, and 5 μL of donor DNA (3 μg total DNA) were combined with 20 μL of cell suspension for transfection in Lonza 4D 96‐well plates using the EH‐115 program of the 4D‐Nucleofector™ system. For a large number of T cells (8–12 × 10^6^ cells), the transfection was scaled up to 120 μL total volume in a Lonza 4D cuvette. In some T cell transfection experiments, AAV6 CAR donor vectors (Virovek, Hayward, CA, USA) were added, using methods as previously described.^31^ In some experiments, to assess the effect of the ROCK inhibitor, Y‐27632 (STEMCELL Technologies, Vancouver, Canada) and the Alt‐R™ HDR Enhancer (Integrated DNA Technologies) on T cell expansion and cytotoxicity following transfection, Y‐27632 was added overnight to the T cell culture media in the absence or presence of Alt‐R™ HDR Enhancer at indicated concentrations.

### 4.6 Flow cytometry

Transfection or transduction efficiency and surface expression of TAG‐72 scFv‐CD3 or TAG‐72 CAR was evaluated by flow cytometry using either GFP or goat anti‐mouse F(ab')_2_ allophycocyanin (APC, Abacus dx, Meadowbrook, QLD, Australia) or anti‐FLAG M2‐fluorescein isothiocyanate (FITC, Sigma‐Aldrich). Frequency of T cell subsets was determined by staining for CD3 phycoerythrin (PE, clone REA613), CD4 VioBlue® (clone VIT4), CD8 VioGreen™ (clone BW135‐80), CCR7 PE‐Vio®770 (clone REA675), and CD45RO APC (clone REA611). All antibodies were acquired from Miltenyi Biotec unless otherwise stated. Staining was performed at 4°C for 15 min. Cells were then washed with fluorescent‐activated cell sorting (FACS) buffer (0.2% (v/v) bovine serum albumin (BSA) (Sigma‐Aldrich) in D‐PBS). Either Viobility™ 405/520 dye (Miltenyi Biotec) or propidium iodide (PI, Sigma‐Aldrich) was used to select for viable cells. Data were acquired using the MACSQuant® analyzer 10 (Miltenyi Biotec), and analysis was subsequently performed using FlowLogic™ (Inivai Technologies, Mentone, VIC, Australia). MFI was used as a measure of surface expression level and in some cases the robust coefficient of variation (R‐CV) was used to measure the dispersion of scFv expression and was calculated as follows: (R‐CV = 100 × 1/2(intensity [at 84.13 percentile] − intensity [at 15.87 percentile])/median).^31^

### 4.7 In vitro T cell cytotoxicity assay

The real‐time cell analysis instrument xCELLigence (ACEA Biosciences, San Diego, CA, USA) was utilized for the assessment of FP T cell or CAR‐T cell function in vitro. Before use, effector cells were transferred to fresh T cell expansion medium or recovery medium comprising 5 ng/mL IL‐7 in basal medium (TexMACS™ supplemented with 5% [v/v] hAb serum) for 12–24 h. T cells were co‐cultured with cancer cell lines at an E:T ratio of either 5:1, 1:1, or 1:5 unless otherwise stated. Target cells were plated for 5–20 h in 96‐well electronic microtiter plates (ACEA Biosciences). Following addition of sorted effector cells, cell impedance was monitored at 15 min intervals over at least 20 h, and in some instances for more than 40 h. All data were normalized to the time of addition of effector cells unless otherwise stated and are presented herein as the arbitrary unit normalized cell index (CI). FP and CAR‐T cell function was calculated as % cytotoxicity = ([normalized CI_target cells alone_ − normalized CI_test_]/normalized CI_target cells alone_) × 100.

### 4.8 Xenograft mouse models

All animal experiments were preapproved by the Monash Medical Centre Animal Ethics Committee (MMCA/2016/61, MMCA/2018/04), and all procedures followed the National Health and Medical Research Council of Australia guidelines for the use and care of experimental animals. In vivo models were established using female 6‐ to 12‐week‐old NSG mice either purchased from Australian BioResources or the Animal Resource Centre. OVCAR‐3 (1 × 10^7^) or Raji (5 × 10^5^) cells were prepared in 100 μL of D‐PBS combined with an equal volume of Matrigel (Corning Life Sciences, Corning, NY, USA) and injected s.c. into the back flank. Mice were randomized into experimental groups (4–5 mice/group). OVCAR‐3 tumors were allowed to reach approximately 100 mm^3^ in size before treatment was commenced. Two injections of FP cells or CAR‐T cells were administered i.v. at 5‐day intervals. Raji tumor bearing mice were treated by a single injection of FP cells or CAR‐T cells (i.v.) 3 days after tumor inoculation. Control mice received NT T cells at comparable dosages. Tumor volume and clinical parameters of animal health were monitored regularly until the experimental endpoint. Tumors were measured using digital calipers, and volumes were calculated using (width^2^ × length)/2(mm^3^). Mice were euthanized when tumor volume reached 1000 mm^3^ or at experiment end by CO_2_ inhalation.

### 4.9 Continuous antigen exposure analysis

To generate CD19 and TAG‐72 dual targeting (CD19/CD3ε + TAG‐72/CD28) FP T cells, a CD19 scFv was knocked‐in at the CD3ε locus and a TAG‐72 scFv was knocked‐in at the CD28 locus as described above. CD19/CD3ε FP T cells were generated in parallel as a control. CD19/CD3ε + TAG‐72/CD28 FP T or CD19/CD3ε FP cells were isolated using the FACS Aria (BD Biosciences, San Jose, CA, USA) as described above. Following isolation, cells were allowed to recover in T cell expansion medium for at least 3 days before subsequent use. The CD19 over‐expressing OVCAR‐3 cells were irradiated (30 Gy), and seeded prior to addition of single antigen targeting or dual targeting FP T cells. FP T cells were moved to fresh irradiated OVCAR‐3 cells daily for 7 days with complete medium changes performed every alternate day. Following 7 days of continued antigen exposure, in vitro cytotoxicity was assessed using xCELLigence.

### 4.10 Statistical analysis

Values represent mean ± SD or *SEM* for at least two to three independent experiments performed in technical duplicate unless otherwise stated. One‐way or two‐way analysis of variance (ANOVA) with Holm–Sidak or Dunnet's test was performed for multiple comparison tests. An unpaired or paired t‐test was performed for comparison of two groups. Significance was defined as *p* ≤ 0.05 and denoted in figures as *p ≤ 0.05, **p ≤ 0.01, ***p ≤ 0.001 or ****p ≤ 0.0001. Analysis was performed using GraphPad Prism 9 software (San Diego, CA, USA).

CRISPR indel analysis, off‐target analysis, Fluorescent Activated Cell Sorting and the Cell lines used are provided in Supporting information.

## 5 CONCLUSIONS

Following optimization of our nonviral CRISPR/Cas9 method, we achieved a high transfection efficiency (≈40%), robust cell recovery and sufficient expansion of FP T cells. FP T cells were able to kill respective cancer targets rapidly in vitro and displayed anti‐tumor activity in vivo in mouse xenograft models. Antigen‐low escape is a significant challenge to cell therapy application. Therefore, by incorporating scFvs into the other CD3 subunits, we were able to generate dual antigen targeting FP T cells using our optimized nonviral CRISPR/Cas9 method to tackle this challenge. Additionally, these dual antigen targeting FP T cells were more resistant to antigen‐induced exhaustion when CD3 and CD28 costimulatory signaling was incorporated. In contrast to viral‐based methods, this method serves as a versatile and alternative way of generating tumor antigen targeting T cells for cancer immunotherapy.

## AUTHOR CONTRIBUTIONS

**Runzhe Shu:** Conceptualization (lead); data curation (equal); formal analysis (equal); methodology (equal); writing – original draft (lead); writing – review and editing (lead). **Maree Hammett:** Data curation (equal); formal analysis (equal); methodology (equal); writing – original draft (equal); writing – review and editing (equal). **Vera Evtimov:** Data curation (equal); formal analysis (equal); methodology (equal); writing – original draft (equal); writing – review and editing (equal). **Aleta Pupovac:** Data curation (equal); writing – original draft (lead); writing – review and editing (lead). **Nhu‐Y Nguyen:** Data curation (equal); formal analysis (equal); methodology (equal). **Rasa Islam:** Formal analysis (equal); methodology (equal). **Junli Zhuang:** Data curation (equal); formal analysis (equal). **Seyeong Lee:** Data curation (equal). **Tae‐hun Kang:** Data curation (equal). **Kyujun Lee:** Formal analysis (equal). **Ian Nisbet:** Supervision (equal); writing – review and editing (equal). **Peter Hudson:** Methodology (equal); writing – review and editing (equal). **Jae Young Lee:** Formal analysis (equal); writing – review and editing (equal). **Richard Boyd:** Funding acquisition (equal); methodology (equal); supervision (equal); writing – review and editing (equal). **Alan Trounson:** Funding acquisition (equal); supervision (equal); writing – review and editing (equal).

## CONFLICT OF INTEREST STATEMENT

The research described in this paper was funded by Cartherics Pty Ltd. All authors, except for SL, TK, KL and JYL are paid employees or advisors of Cartherics and hold options and/or equity in the company. SL, TK, KL and JYL are employees of ToolGen Inc.

### PEER REVIEW

The peer review history for this article is available at https://www.webofscience.com/api/gateway/wos/peer-review/10.1002/btm2.10571.

## Supporting information


**Data S1.** Supporting information.

## Data Availability

Datasets are available upon reasonable request.

## References

[1] Grupp SA , Kalos M , Barrett D , et al. Chimeric antigen receptor‐modified T cells for acute lymphoid leukemia. N Engl J Med. 2013;368(16):1509‐1518. 23527958 10.1056/NEJMoa1215134 PMC4058440

[2] Wang LL , Janes ME , Kumbhojkar N , et al. Cell therapies in the clinic. Bioeng Transl Med. 2021;6(2):e10214. 34027097 10.1002/btm2.10214 PMC8126820

[3] Bulcha JT , Wang Y , Ma H , Tai PWL , Gao G . Viral vector platforms within the gene therapy landscape. Signal Transduct Target Ther. 2021;6(1):53. 33558455 10.1038/s41392-021-00487-6 PMC7868676

[4] Genovese G , Kahler AK , Handsaker RE , et al. Clonal hematopoiesis and blood‐cancer risk inferred from blood DNA sequence. N Engl J Med. 2014;371(26):2477‐2487. 25426838 10.1056/NEJMoa1409405 PMC4290021

[5] Escors D , Breckpot K . Lentiviral vectors in gene therapy: their current status and future potential. Arch Immunol Ther Exp (Warsz). 2010;58(2):107‐119. 20143172 10.1007/s00005-010-0063-4 PMC2837622

[6] Comisel R‐M , Kara B , Fiesser FH , Farid SS . Lentiviral vector bioprocess economics for cell and gene therapy commercialization. Biochem Eng J. 2021;167:107868.

[7] Kebriaei P , Singh H , Huls MH , et al. Phase I trials using sleeping beauty to generate CD19‐specific CAR T cells. J Clin Invest. 2016;126(9):3363‐3376. 27482888 10.1172/JCI86721 PMC5004935

[8] Bishop DC , Clancy LE , Simms R , et al. Development of CAR T‐cell lymphoma in 2 of 10 patients effectively treated with piggyBac‐modified CD19 CAR T cells. Blood. 2021;138(16):1504‐1509. 34010392 10.1182/blood.2021010813

[9] Micklethwaite KP , Gowrishankar K , Gloss BS , et al. Investigation of product‐derived lymphoma following infusion of piggyBac‐modified CD19 chimeric antigen receptor T cells. Blood. 2021;138(16):1391‐1405. 33974080 10.1182/blood.2021010858 PMC8532197

[10] Doudna JA , Charpentier E . Genome editing. The new frontier of genome engineering with CRISPR‐Cas9. Science. 2014;346(6213):1258096. 25430774 10.1126/science.1258096

[11] Sharma G , Sharma AR , Bhattacharya M , Lee SS , Chakraborty C . CRISPR‐Cas9: a preclinical and clinical perspective for the treatment of human diseases. Mol Ther. 2021;29(2):571‐586. 33238136 10.1016/j.ymthe.2020.09.028 PMC7854284

[12] Li Y , Glass Z , Huang M , Chen ZY , Xu Q . Ex vivo cell‐based CRISPR/Cas9 genome editing for therapeutic applications. Biomaterials. 2020;234(3):119711. 31945616 10.1016/j.biomaterials.2019.119711 PMC7035593

[13] Cornu TI , Mussolino C , Cathomen T . Refining strategies to translate genome editing to the clinic. Nat Med. 2017;23(4):415‐423. 28388605 10.1038/nm.4313

[14] Sadelain M , Riviere I , Riddell S . Therapeutic T cell engineering. Nature. 2017;545(7655):423‐431. 28541315 10.1038/nature22395 PMC5632949

[15] Shafer P , Kelly LM , Hoyos V . Cancer therapy with TCR‐engineered T cells: current strategies, challenges, and prospects. Front Immunol. 2022;13:835762. 35309357 10.3389/fimmu.2022.835762 PMC8928448

[16] Labrijn AF , Janmaat ML , Reichert JM , Parren P . Bispecific antibodies: a mechanistic review of the pipeline. Nat Rev Drug Discov. 2019;18(8):585‐608. 31175342 10.1038/s41573-019-0028-1

[17] Evtimov V , Boyd R , Nisbet I , Prince M , Trounson A . T Cell Disease Treatment Targeting TAG‐72. PCT Patent WO2019161439 filed 18 February 2019 and published 29 August 2019; 2019.

[18] Nagle JA , Wilbur DC , Pitman MB . Cytomorphology of gastric and duodenal epithelium and reactivity to B72.3: a baseline for comparison to pancreatic lesions aspirated by EUS‐FNAB. Diagn Cytopathol. 2005;33(6):381‐386. 16299750 10.1002/dc.20343

[19] Julien S , Videira PA , Delannoy P . Sialyl‐tn in cancer: (how) did we miss the target? Biomolecules. 2012;2(4):435‐466. 24970145 10.3390/biom2040435 PMC4030860

[20] Minnix M , Li L , Yazaki P , et al. Improved targeting of an anti‐TAG‐72 antibody drug conjugate for the treatment of ovarian cancer. Cancer Med. 2020;9(13):4756‐4767. 32368864 10.1002/cam4.3078 PMC7333846

[21] Scott AM , Akhurst T , Lee FT , et al. First clinical study of a pegylated diabody (124)I‐labeled PEG‐AVP0458 in patients with tumor‐associated glycoprotein 72 positive cancers. Theranostics. 2020;10(25):11404‐11415. 33052222 10.7150/thno.49422 PMC7545991

[22] Murad JP , Kozlowska AK , Lee HJ , et al. Effective targeting of TAG72(+) peritoneal ovarian tumors via regional delivery of CAR‐engineered T cells. Front Immunol. 2018;9:2268. 30510550 10.3389/fimmu.2018.02268 PMC6254427

[23] Mali P , Yang L , Esvelt KM , et al. RNA‐guided human genome engineering via Cas9. Science. 2013;339(6121):823‐826. 23287722 10.1126/science.1232033 PMC3712628

[24] Shu R , Evtimov VJ , Hammett MV , et al. Engineered CAR‐T cells targeting TAG‐72 and CD47 in ovarian cancer. Mol Ther Oncolytics. 2021;20(3):325‐341. 33614914 10.1016/j.omto.2021.01.002 PMC7868933

[25] Wang X , Ma C , Rodriguez Labrada R , et al. Recent advances in lentiviral vectors for gene therapy. Sci China Life Sci. 2021;64(11):1842‐1857. 34708326 10.1007/s11427-021-1952-5

[26] Hong M , Clubb JD , Chen YY . Engineering CAR‐T cells for next‐generation cancer therapy. Cancer Cell. 2020;38(4):473‐488. 32735779 10.1016/j.ccell.2020.07.005

[27] Yu C , Liu Y , Ma T , et al. Small molecules enhance CRISPR genome editing in pluripotent stem cells. Cell Stem Cell. 2015;16(2):142‐147. 25658371 10.1016/j.stem.2015.01.003 PMC4461869

[28] Claassen DA , Desler MM , Rizzino A . ROCK inhibition enhances the recovery and growth of cryopreserved human embryonic stem cells and human induced pluripotent stem cells. Mol Reprod Dev. 2009;76(8):722‐732. 19235204 10.1002/mrd.21021 PMC3257892

[29] Alarcon B , Ley SC , Sanchez‐Madrid F , et al. The CD3‐gamma and CD3‐delta subunits of the T cell antigen receptor can be expressed within distinct functional TCR/CD3 complexes. EMBO J. 1991;10(4):903‐912. 1826255 10.1002/j.1460-2075.1991.tb08023.x PMC452733

[30] San Jose E , Sahuquillo AG , Bragado R , Alarcon B . Assembly of the TCR/CD3 complex: CD3 epsilon/delta and CD3 epsilon/gamma dimers associate indistinctly with both TCR alpha and TCR beta chains. Evidence for a double TCR heterodimer model. Eur J Immunol. 1998;28(1):12‐21. 9485181 10.1002/(SICI)1521-4141(199801)28:01<12::AID-IMMU12>3.0.CO;2-9

[31] Eyquem J , Mansilla‐Soto J , Giavridis T , et al. Targeting a CAR to the TRAC locus with CRISPR/Cas9 enhances tumour rejection. Nature. 2017;543(7643):113‐117. 28225754 10.1038/nature21405 PMC5558614

[32] Han X , Wang Y , Wei J , Han W . Multi‐antigen‐targeted chimeric antigen receptor T cells for cancer therapy. J Hematol Oncol. 2019;12(1):128. 31783889 10.1186/s13045-019-0813-7 PMC6884912

[33] Cappell KM , Kochenderfer JN . A comparison of chimeric antigen receptors containing CD28 versus 4‐1BB costimulatory domains. Nat Rev Clin Oncol. 2021;18(11):715‐727. 34230645 10.1038/s41571-021-00530-z

[34] Meng X , Wu TG , Lou QY , et al. Optimization of CRISPR‐Cas system for clinical cancer therapy. Bioeng Transl Med. 2023;8(2):e10474. 36925702 10.1002/btm2.10474 PMC10013785

[35] Roth TL , Puig‐Saus C , Yu R , et al. Reprogramming human T cell function and specificity with non‐viral genome targeting. Nature. 2018;559(7714):405‐409. 29995861 10.1038/s41586-018-0326-5 PMC6239417

[36] Zhang J , Hu Y , Yang J , et al. Non‐viral, specifically targeted CAR‐T cells achieve high safety and efficacy in B‐NHL. Nature. 2022;609(7926):369‐374. 36045296 10.1038/s41586-022-05140-y PMC9452296

[37] Harris E , Elmer JJ . Optimization of electroporation and other non‐viral gene delivery strategies for T cells. Biotechnol Prog. 2021;37(1):e3066. 32808434 10.1002/btpr.3066

[38] Paix A , Folkmann A , Goldman DH , et al. Precision genome editing using synthesis‐dependent repair of Cas9‐induced DNA breaks. Proc Natl Acad Sci U S A. 2017;114(50):E10745‐E10754. 29183983 10.1073/pnas.1711979114 PMC5740635

[39] Chen Y , Chen Y , Yin W , et al. The regulation of DOCK family proteins on T and B cells. J Leukoc Biol. 2021;109(2):383‐394. 32542827 10.1002/JLB.1MR0520-221RR

[40] Feucht J , Sun J , Eyquem J , et al. Calibration of CAR activation potential directs alternative T cell fates and therapeutic potency. Nat Med. 2019;25(1):82‐88. 30559421 10.1038/s41591-018-0290-5 PMC6532069

[41] Gomes‐Silva D , Mukherjee M , Srinivasan M , et al. Tonic 4‐1BB Costimulation in chimeric antigen receptors impedes T cell survival and is vector‐dependent. Cell Rep. 2017;21(1):17‐26. 28978471 10.1016/j.celrep.2017.09.015 PMC5645034

[42] Hu JH , Davis KM , Liu DR . Chemical biology approaches to Genome editing: understanding, controlling, and delivering programmable nucleases. Cell Chem Biol. 2016;23(1):57‐73. 26933736 10.1016/j.chembiol.2015.12.009

[43] Chu VT , Weber T , Wefers B , et al. Increasing the efficiency of homology‐directed repair for CRISPR‐Cas9‐induced precise gene editing in mammalian cells. Nat Biotechnol. 2015;33(5):543‐548. 25803306 10.1038/nbt.3198

[44] Maruyama T , Dougan SK , Truttmann MC , Bilate AM , Ingram JR , Ploegh HL . Increasing the efficiency of precise genome editing with CRISPR‐Cas9 by inhibition of nonhomologous end joining. Nat Biotechnol. 2015;33(5):538‐542. 25798939 10.1038/nbt.3190 PMC4618510

[45] Robert F , Barbeau M , Ethier S , Dostie J , Pelletier J . Pharmacological inhibition of DNA‐PK stimulates Cas9‐mediated genome editing. Genome Med. 2015;7(8):93. 26307031 10.1186/s13073-015-0215-6 PMC4550049

[46] Zhang H , Zhao P , Huang H . Engineering better chimeric antigen receptor T cells. Exp Hematol Oncol. 2020;9(1):34. 33292660 10.1186/s40164-020-00190-2 PMC7709221

[47] Sterner RC , Sterner RM . CAR‐T cell therapy: current limitations and potential strategies. Blood Cancer J. 2021;11(4):69. 33824268 10.1038/s41408-021-00459-7 PMC8024391

[48] Xu Y , Yang Z , Horan LH , et al. A novel antibody‐TCR (AbTCR) platform combines Fab‐based antigen recognition with gamma/delta‐TCR signaling to facilitate T‐cell cytotoxicity with low cytokine release. Cell Discov. 2018;4:62. 30479831 10.1038/s41421-018-0066-6 PMC6242878

[49] Baeuerle PA , Ding J , Patel E , et al. Synthetic TRuC receptors engaging the complete T cell receptor for potent anti‐tumor response. Nat Commun. 2019;10(1):2087. 31064990 10.1038/s41467-019-10097-0 PMC6504948

[50] Shu R , Trounson A , Boyd R, Nisbet I , Boyd N , Evtimov V . Method for Providing Immune Cells with Enhanced Function. PCT Patent WO2021097521A1 filed 18 November 2020 and published 27 May 2021; 2021.

[51] Shu R , Trounson A , Nisbet I , Boyd N , Boyd R , Evtimov V . Immune Cells Expressing Modified Cell Receptors and Methods of Making. PCT Patent WO2021022327A1 filed 4 August 2020 and published 11 February 2021; 2021.

[52] Boyd R , Trounson A , Kawamoto H , Hudson PJ , Shu R . Genetically Modified Cells and Uses Thereof. US Patent US11400145B2 filed 23 November 2016 and published 2 August 2022; 2022.

[53] Shu R , Wong W , Ma QH , et al. APP intracellular domain acts as a transcriptional regulator of miR‐663 suppressing neuronal differentiation. Cell Death Dis. 2015;6(2):e1651. 25695604 10.1038/cddis.2015.10 PMC4669786

