# Supplementary material for: Engineering T cell receptor fusion proteins using nonviral CRISPR/Cas9 genome editing for cancer immunotherapy
Source: Bioeng Transl Med. 2023 Jul 10;8(6):e10571. doi: 10.1002/btm2.10571 (PMC10658519; doi:10.1002/btm2.10571)
Supplement: Supplementary file 1 — Data S1. Supporting information. [file BTM2-8-e10571-s001.docx]

**Supporting Information: List of figures and tables**

**Figure S1:** CRISPR/Cas9 KI of GFP or CAR expression cassette into activated human T cells using plasmid or dsDNA.

**Figure S2:** KI efficiency of dsDNA is improved through the reduction of homology arm length.

**Figure S3:** CD3ε gRNA-1 introduces indels at the CD3ε gene N-terminus at a higher efficiency compared to CD3ε gRNA-2.

**Figure S4:** *In silico* off-target analysis of CD3ε gRNA-1 in primary human T cells.

**Figure S5:** TAG-72/CD3ε FP T cells derived from a second donor demonstrates anti-tumor activity *in vivo*.

**Figure S6:** TAG-72/CD28 FP T cells do not kill TAG-72 positive tumor targets *in vitro*.

**Table S1:** Potential off-target sites of CD3ε gRNA-1 identified from *in silico* analysis (mismatches in red).

**Table S2**: Primers utilized in the off-target analysis.

**Supporting Information: Methods**

**CRISPR insertions and deletions (indel) analysis**

The efficacy and the mutation spectrum of CRISPR/Cas9 genome editing was analyzed by the Inference of CRISPR Edits (ICE) assay.^1^ Genomic DNA was extracted from cells 4 days after electroporation using the NucleoSpin^®^ Blood DNA Mini Kit (MACHEREY-NAGEL, Duren, Germany) following the manufacturer's instructions. PCR amplicons spanning the gRNA genomic target sites were generated using the Q5^®^ High-Fidelity Taq polymerase (New England Biolabs, Ipswich, MA, USA). For analyzing genetic modification frequencies using ICE, the purified PCR products were Sanger-sequenced and the sequence chromatograms analyzed using the online Synthego ICE Analysis tool (https://ice.synthego.com/#/) .

**Off-target analysis**

Potential off-target sites were identified *in silico* using the online Cas-OFFinder tool (www.rgenome.net).^2^ Targeted deep sequencing was performed as described previously.^3^ Data from deep sequencing were analyzed using the online Cas-Analyzer tool (www.rgenome.net).^2^ Indels in the region 3 bp upstream from the protospacer-adjacent motif (PAM) sequence were considered as editing resulting from Cas9. Primer lists utilized in this study are provided in **Table S2**. Due to high GC content and repeat nucleotides, the Off11 site was not analyzed (N/A).

**Fluorescent-Activated Cell Sorting (FACS)**

FP or CAR-positive cells were isolated following 10–13 days in culture using either GFP or F(ab′)_2_-APC or FLAG-FITC as a reporter for CAR expression. Viable cells were selected for using Viobility^™^ 405/452 dye. Staining was performed at 4°C for 15 min in FACS buffer. Briefly, cells were resuspended in FACS buffer and incubated with primary antibodies as previously described. Sorts were performed using the FACSAria^™^ Fusion droplet sorter (BD Biosciences, San Jose, CA, USA). Resultant cells were maintained in complete T cell expansion medium until required.

**Cell lines**

All cell lines (ovarian cancer cell line OVCAR-3 (HTB-161); ovarian cancer cell line derived from ascites MES-OV (CRL-3272), HeLa (CCL-2) and Raji (CCL-86) cells) were acquired from the American Type Culture Collection (ATCC, Manassas, VA, USA) and maintained using recommended culture conditions. OVCAR-3 and HeLa cells were transduced using human CD19 lentivirus and then sorted using the FACSAria^™^ Fusion droplet sorter (BD Biosciences, San Jose, CA, USA) to generate CD19 overexpressing stable cell lines.

**References**

**1.** Conant D, Hsiau T, Rossi N, et al. Inference of CRISPR Edits from Sanger Trace Data. *CRISPR J.* Feb 2022;5(1):123-130.

**2.** Park J, Lim K, Kim JS, Bae S. Cas-analyzer: an online tool for assessing genome editing results using NGS data. *Bioinformatics.* Jan 15 2017;33(2):286-288.

**3.** Lee JS, Lee JY, Song DW, et al. Targeted PMP22 TATA-box editing by CRISPR/Cas9 reduces demyelinating neuropathy of Charcot-Marie-Tooth disease type 1A in mice. *Nucleic Acids Res.* Jan 10 2020;48(1):130-140.


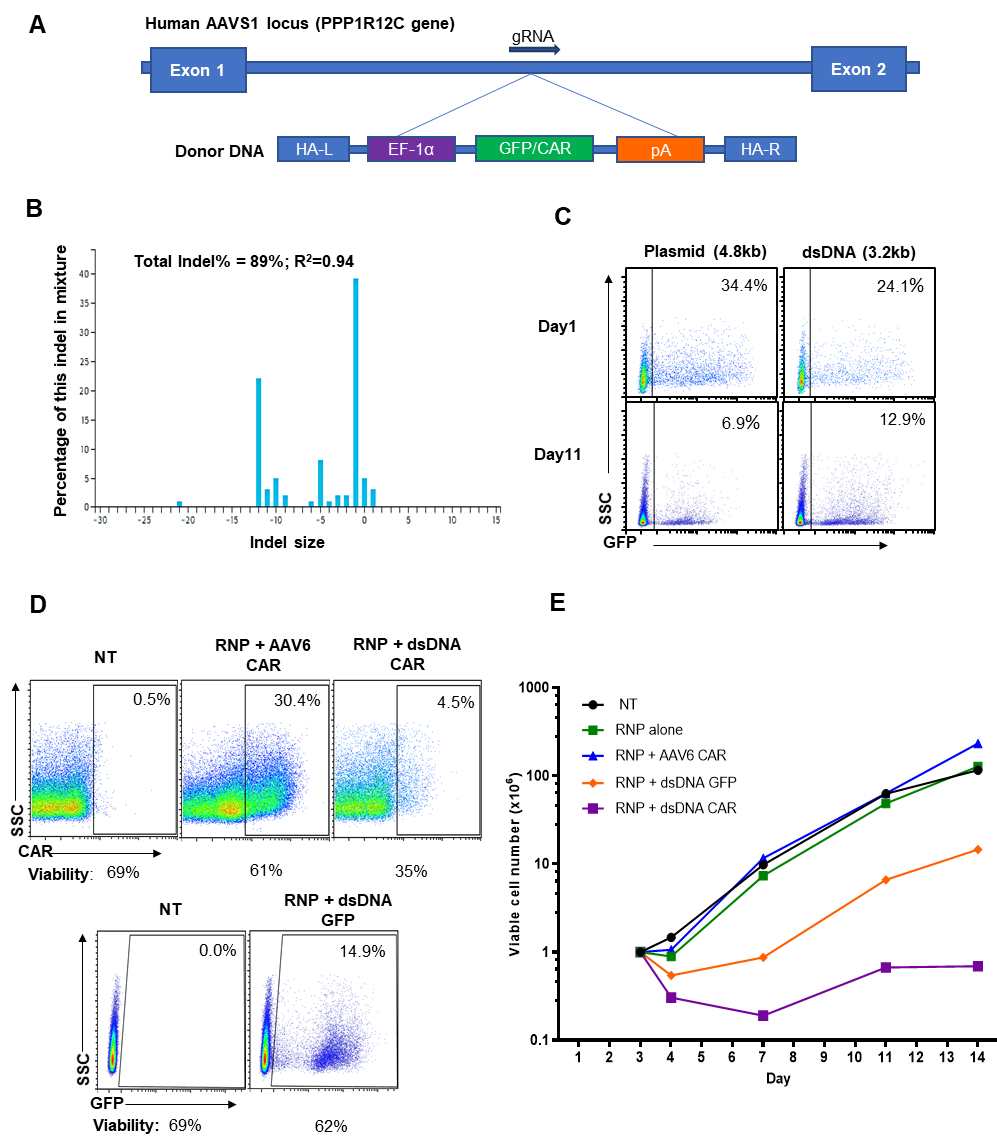


**Figure S1:** CRISPR/Cas9 KI of GFP or CAR expression cassettes into activated human T cells using plasmid or dsDNA. (**A**) Schematic of the GFP or CAR expression cassette used for KI of DNA into the AAVS1 (PPP1R12C gene) locus. The donor DNA was comprised of two homologous arms (HA-L and HA-R), the human EF-1α constitutive promoter, a poly A terminator and the coding sequence of GFP or TAG-72 CAR. Co-transfection of AAVS1 gRNA (sgRNA-t2) formed Cas9 ribonucleoprotein (RNP) introduces the GFP or TAG-72 CAR expression cassette into the AAVS1 locus through HDR. (**B**) ICE analysis showed that sgRNA-t2 achieved high efficiency of gene editing (%). (**C**) GFP transient transfection efficiency (%, Day 1) and GFP stable integration efficiency (%, Day 11) (figures embedded within the pseudo-colored dot plot) using a 4.8 kb plasmid or a 3.2 kb dsDNA fragment was determined by flow cytometry 1 and 11 days after transfection respectively. (**D**) Transfection efficiency (%) of the TAG-72 CAR (3.1kb) or GFP (2.4kb) dsDNA (figure embedded within the pseudo-colored dot plot) and respective viability (%) (denoted below each respective pseudo-colored dot plot) in human T cells are shown 10 days following activation. AAV6 transduction was performed as a positive control. (**E**) Expansion of non-transfected (NT), AAVS1 RNP only transfected, AAV6 transduced TAG-72 CAR, or non-viral transfected GFP and TAG-72 CAR-T cells were tracked for 14 days following activation. Representative data are shown from two independent experiments using T cells from different healthy donors.


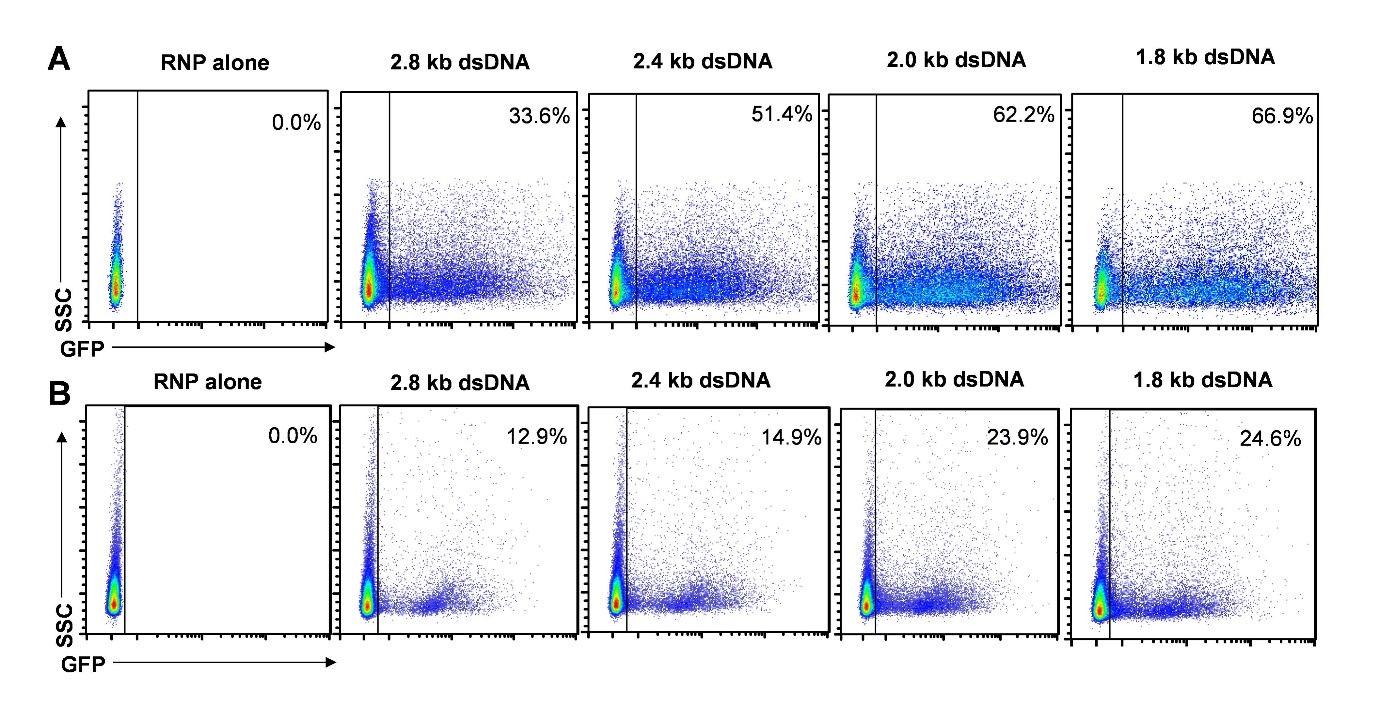


**Figure S2:** KI efficiency of dsDNA is improved through the reduction of homology arm length. AAVS1 gRNA formed Cas9 RNP and GFP-tagged donor dsDNA of differing lengths (as indicated) were co-transfected into the AAVS1 locus. Flow cytometry analysis of GFP expression was performed (**A**) 1 day and (**B**) 7 days after transfection, demonstrating the KI efficiency of the GFP cassette. T cells transfected with AAVS1 Cas9 RNP alone were utilized as a control. Representative data are shown from two independent experiments using T cells from different healthy donors.

**
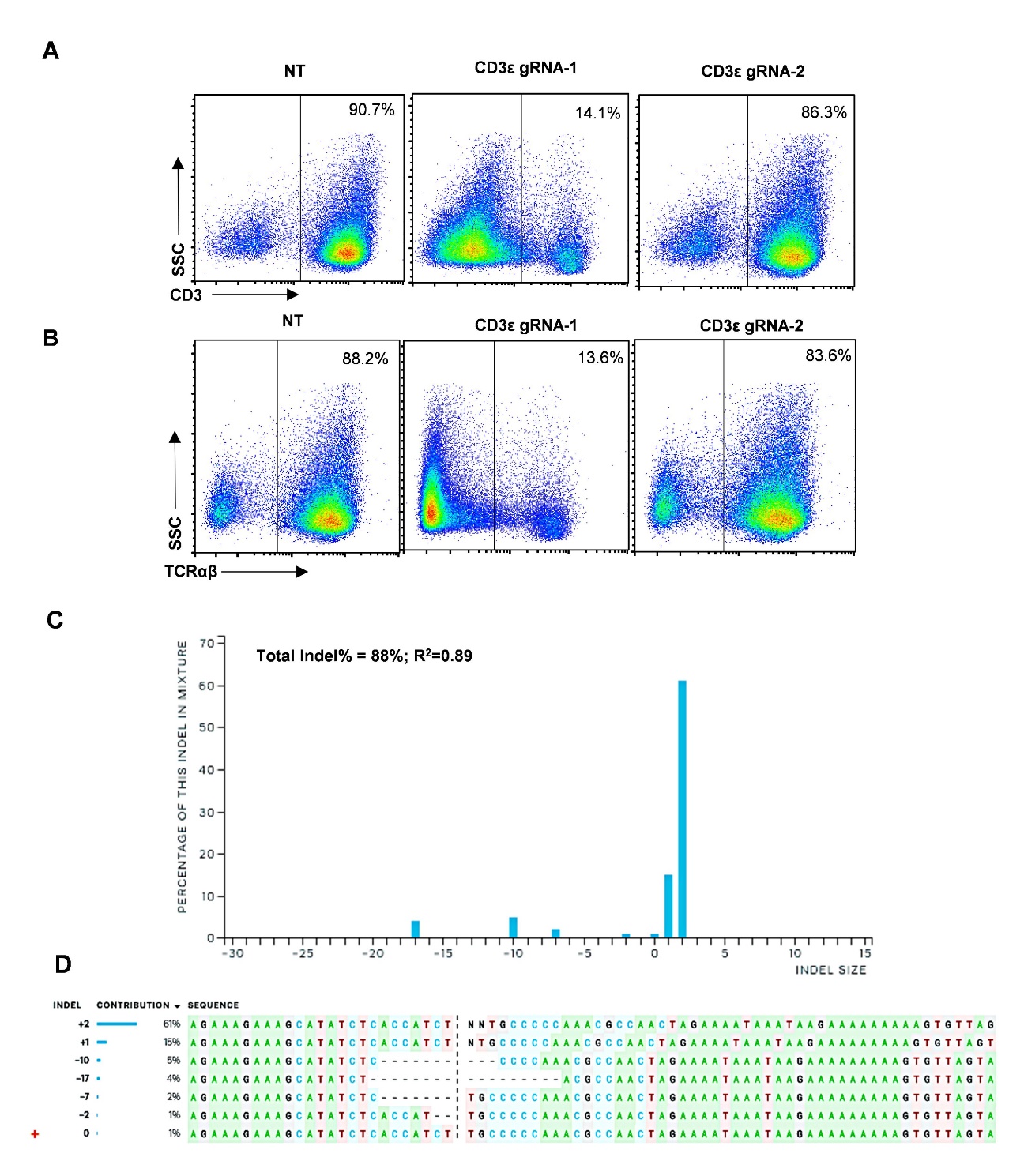
**

**Figure S3:** CD3ε gRNA-1 introduces indels at the CD3ε gene N-terminus at a higher efficiency compared to CD3ε gRNA-2. The surface expression of (**A**) CD3 and (**B**) TCRαβ in T cells 4 days after CD3ε RNP co-transfection with CD3ε gRNA-1 or CD3ε gRNA-2 was analyzed by flow cytometry. Non-transfected (NT) T cells were included as a control. Transfection of CD3ε gRNA-1 formed Cas9 RNP introduces indels into CD3ε Exon 3. (**C** and **D**) Frequency of indels was assessed by ICE analysis. Representative data are shown using T cells from one healthy donor.


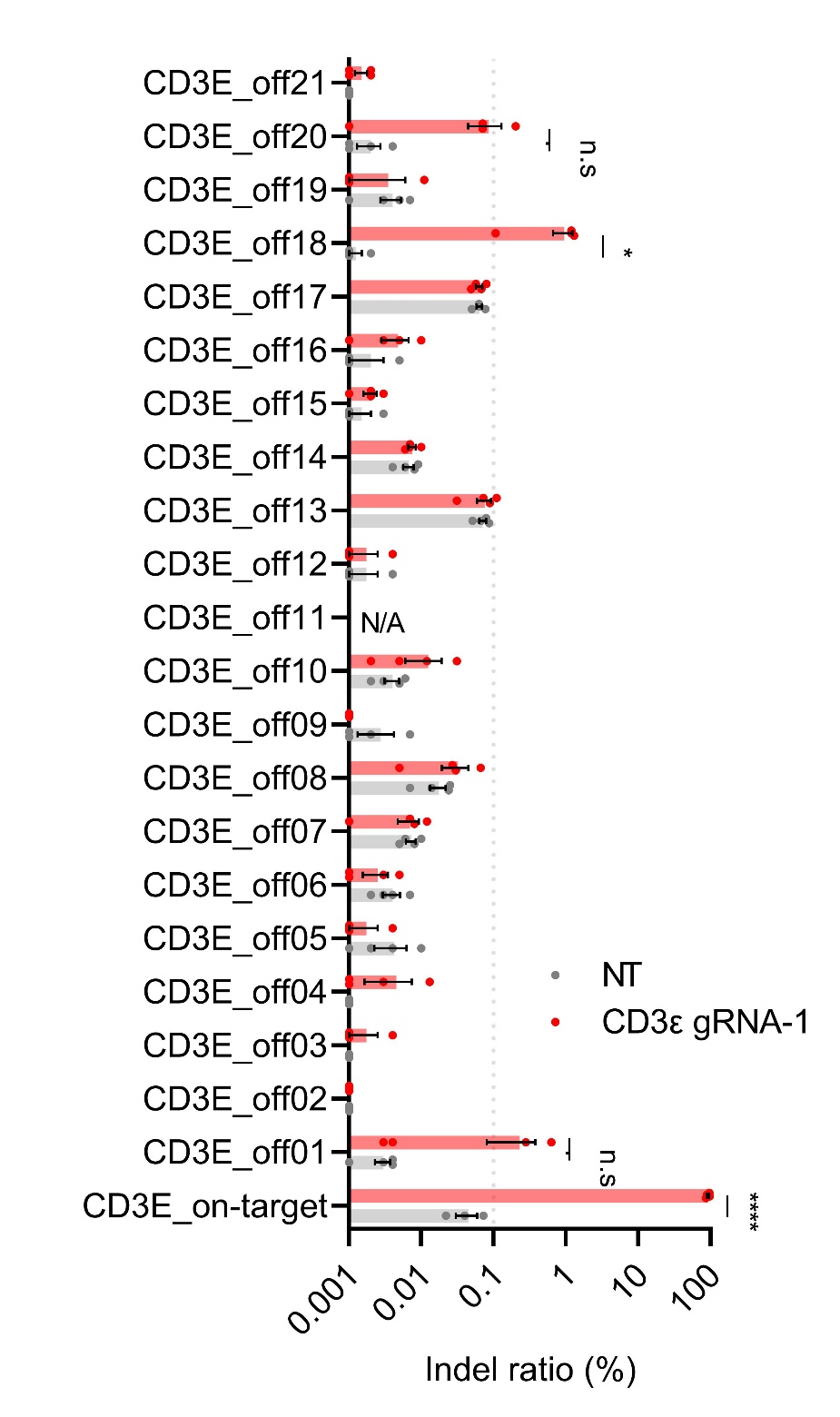


**Figure S4. *In silico* off-target analysis of CD3**ε **gRNA-1 in primary human T cells.** Indel frequencies in primary human T cells, determined by targeted deep sequencing at CD3ε gRNA-1 on-target and off-target sites identified by *in silico* off-target analysis. Non-transfected (NT) T cells were used as a control. Data are represented as mean ± SEM from 4 independent experiments using T cells from different healthy donors. * p ≤ 0.05, ****p ≤ 0.001 using a t-test.


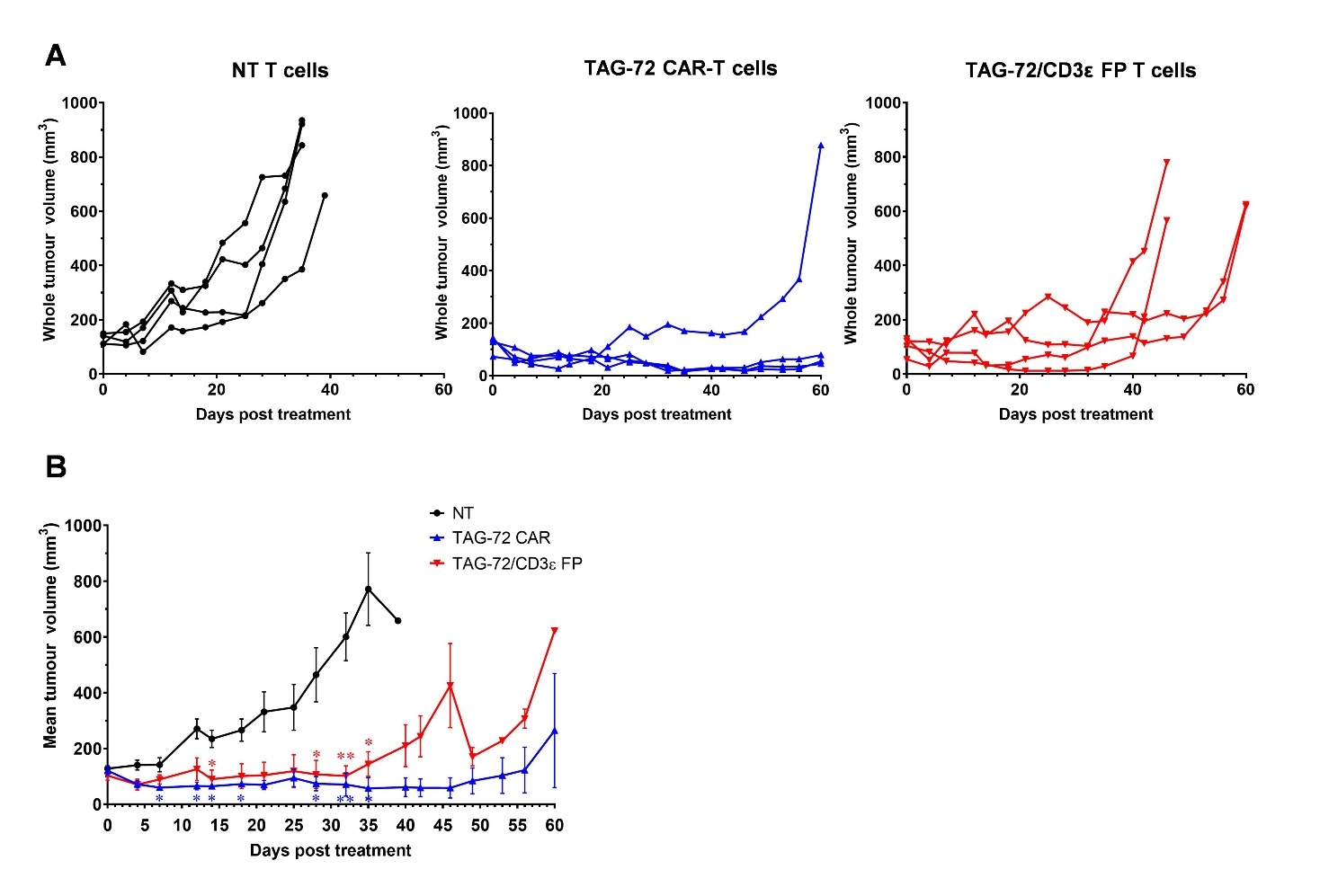


**Figure S5:** TAG-72/CD3ε FP T cells derived from a second donor demonstrates anti-tumor activity *in vivo*. (**A, B**) NSG mice bearing OVCAR-3 derived tumors were treated at Day 0 and Day 5 with 5x10^6^ TAG-72/CD3ε FP, TAG-72 CAR-T or non-transfected (NT) T cells by i.v. injection when tumor volume reached approximately 100mm^3^. Tumor volume was monitored for 60 days. (**B**) Data are represented as mean ± SEM (n=4, T cells from one healthy donor, *p ≤ 0.05, **p ≤ 0.01, using a two-way ANOVA).


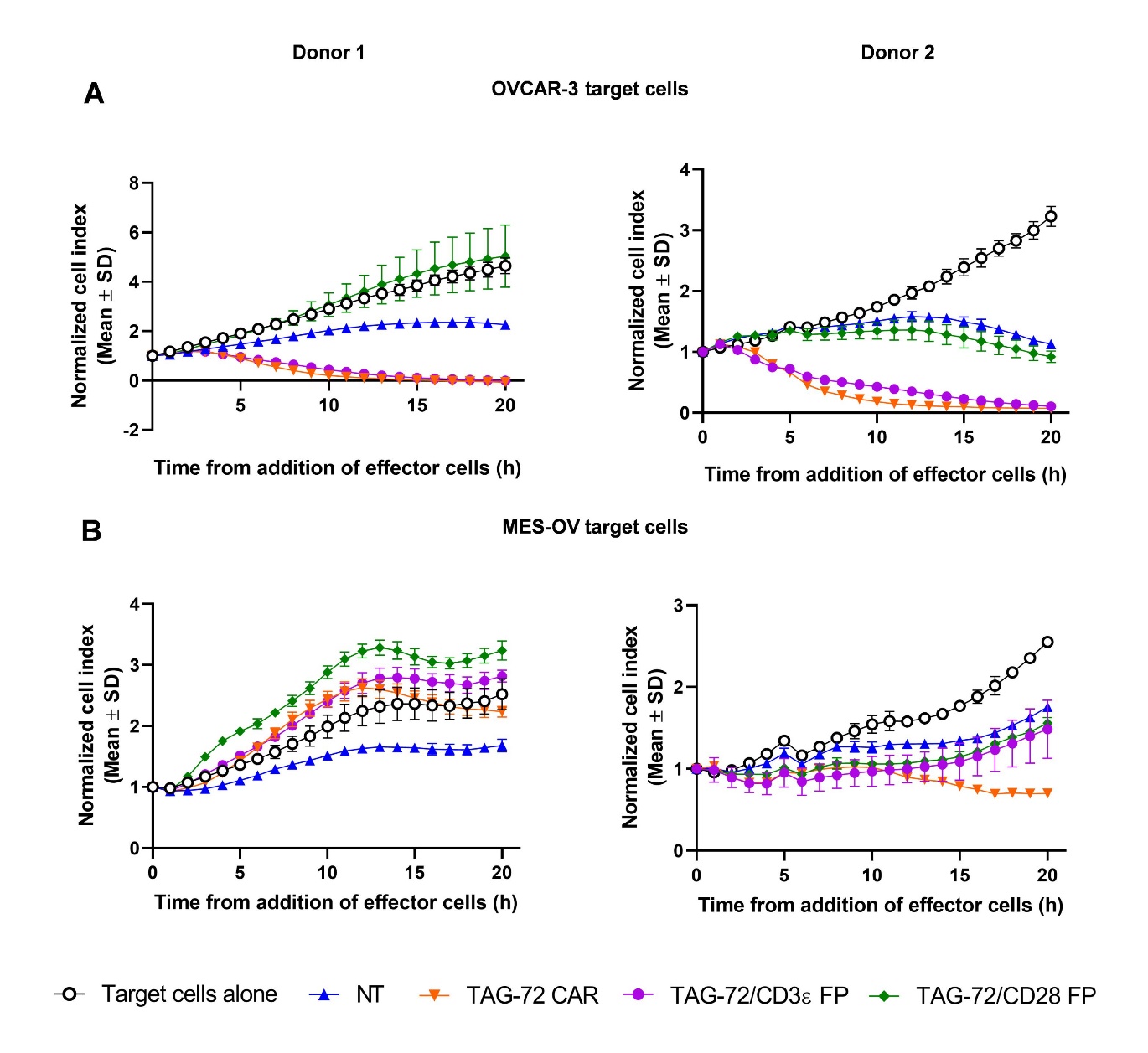


**Figure S6**. TAG-72/CD28 FP T cells do not kill TAG-72 positive tumor targets *in vitro*. (**A**) TAG-72^hi^ OVCAR-3 or (**B**) TAG-72^low^ MES-OV target cells were co-cultured with TAG-72 CAR-T cells, TAG-72/CD3ε FP T cells, or TAG-72/CD28 FP T cells at an E:T ratio of 5:1 and cell impedance (normalized cell index) was monitored over 20h. In parallel, non-transfected (NT) T cells were utilized as controls and target cell proliferation under normal growth conditions was also monitored. Data are represented as mean ± SD of technical duplicates or triplicates, where CAR and FP T cells were generated using T cells from two independent healthy donors (Donor 1 and Donor 2).

**Table S1:** Potential off-target sites of CD3ε gRNA-1 identified from *in silico* analysis (mismatches in red).

| **Name** | **Chromosome** | **Position** | **Mismatches** | **Location information** | **DNA** |
| --- | --- | --- | --- | --- | --- |
| CD3E_on-target | chr11 | 118,307,287 | 0 | CD3E_exon | GTTGGCGTTTGGGGGCAAGATGG |
| CD3E_off01_in silico | chr8 | 39,053,690 | 3 | ADAM9_intron | GTgGGaGTTTGGGGGtAAGAAGG |
| CD3E_off02_in silico | chr15 | 40,354,705 | 3 | PHGR1_intron | GTTGGaGTTTGGGaGCAAGcCAG |
| CD3E_off03_in silico | chr1 | 5,814,293 | 3 | intergenic | GTTGGgGTTTGGGGcgAAGAGGG |
| CD3E_off04_in silico | chr1 | 227,061,213 | 3 | CDC42BPA_intron | GTTtGgtTTTGGGGGCAAGAAGG |
| CD3E_off05_in silico | chr22 | 25,901,245 | 3 | MYO18B_intron | tTTGGCcTTTGGtGGCAAGAAAG |
| CD3E_off06_in silico | chr7 | 11,447,915 | 3 | THSD7A_intron | GTTGGaGTTTGGGGtCAgGAAGG |
| CD3E_off07_in silico | chr7 | 143,265,347 | 3 | GSTK1, TMEM139-AS1_intron | GTTGGCGTTTGGGGGtAAagAAG |
| CD3E_off08_in silico | chr7 | 149,630,461 | 3 | intergenic | GcTGGgGTTTGGGGGCAAcATGG |
| CD3E_off09_in silico | chr2 | 27,208,788 | 3 | SLC5A6_intron | GTTGGaGTTTGGGGGCcAtAGGG |
| CD3E_off10_in silico | chr4 | 7,271,835 | 3 | SORCS2_intron | GTgGGtGTTTGGGGGCAgGAAGG |
| CD3E_off11_in silico | chr17 | 56,593,584 | 3 | intergenic_NOG upstream | GTTGttGTTTGGGGGgAAGAAGG |
| CD3E_off12_in silico | chrX | 42,166,978 | 3 | intergenic | GTTGGaGgTTGGtGGCAAGATGG |
| CD3E_off13_in silico | chrX | 144,242,791 | 3 | intergenic | GTTGGCGTTTGGGGGaggGAGAG |
| CD3E_off14_in silico | chr6 | 34,125,799 | 3 | GRM4_intron | GTgGGgGTTTGGGGGaAAGAGAG |
| CD3E_off15_in silico | chr11 | 44,412,344 | 3 | intergenic | GTTGGCtTaTGGtGGCAAGAGGG |
| CD3E_off16_in silico | chr11 | 73,067,327 | 3 | FCHSD2_intron | GTTGGgGgTTGGGGGCtAGAGGG |
| CD3E_off17_in silico | chr11 | 112,920,185 | 3 | intergenic | GTTGGaGaTgGGGGGCAAGAAAG |
| CD3E_off18_in silico | chr10 | 127,268,787 | 3 | DOCK1_intron | GgTGGtGTTTGGGaGCAAGATGG |
| CD3E_off19_in silico | chr18 | 2,686,122 | 3 | SMCHD1_intron | tTTGGgGTTTGGGGGgAAGAGAG |
| CD3E_off20_in silico | chr18 | 6,098,380 | 3 | L3MBTL4_intron | aTTGGCGTTTGGGaaCAAGAGGG |
| CD3E_off21_in silico | chr3 | 160,203,892 | 3 | IL12A-AS1_intron | GTTGGCcTTTGGtGGCAgGAGAG |

**Table S2:** Primers utilized in the off-target analysis.

| **Name** | **Forward primer** | **Reverse primer** |
| --- | --- | --- |
| CD3E_on-target | CTGCAACACAGCCCTTTTTCT | AGGCCTAAGAGAAAGCTCCA |
| CD3E_off01 | TGTACTTTTCACCCAGTTTTCCT | CCTTGCTTGGCTTCTCTCAC |
| CD3E_off02 | AACTGAGTCTGCTGGTGGTAG | CCTGTGCTATGCTTTTCTGCT |
| CD3E_off03 | CACACACTTTATGCCTCTGGC | GCTTCATTGAAAGCCACTGCT |
| CD3E_off04 | AGCTAGAGGAAGAGTTGGAGTTG | GGATTAGAAGGCCCGGAGAG |
| CD3E_off05 | TTGCCAAGCCTCAGTAAGGT | AAAGCTCCTGAGCTGTTCCA |
| CD3E_off06 | GTGTTTGGAGAGGGACTGGA | CTTCCCATGCCAATGCAGTTT |
| CD3E_off07 | GCCTTCCTGCTGTCTTCTCTT | GTGCCCTGAATCACAGACAT |
| CD3E_off08 | TGAATGTGGAGAGGAGAGCG | CAGCTTTCCCGTGAAGCCAT |
| CD3E_off09 | CGCACAGAGTGCCAAGAGTA | GGGTGAAGCTCAGCAGTCAT |
| CD3E_off10 | AATGATGGCATGAGTGGGCT | AGGACTCCTTAGCGAACACC |
| CD3E_off11 | N/A | N/A |
| CD3E_off12 | GGTGTGTGGTAGGGGATAGA | CCTCAGTGGCAGTGTTCCA |
| CD3E_off13 | CATGGATGTAGGTGGAAGCC | GATCATCTCATGAAATAGGTGTTAAGC |
| CD3E_off14 | CAGAGGATTGGCTGCCAGTAG | CGAAGTAGGTCTTGCTGAGGG |
| CD3E_off15 | ACTGGTATTACTGTGTTGGAGTT | ATAGTTGAGGGTAGCCAGACG |
| CD3E_off16 | CTCATGGACATAGGGAGGGG | CAGAGGGGAAGTTCAAATTGTTT |
| CD3E_off17 | CTTTCTCGTCCTAGAATGC | TCAGCTCTTAGTTACTCTCC |
| CD3E_off18 | TGGAGGTGGTTTCCCCAAGT | CCCTGAGAACAGGCTCTTGC |
| CD3E_off19 | TTTTTGAAAGTCAAGCTCAAACAG | TGCATGCAAAATCTGAGTCCC |
| CD3E_off20 | TGCAGCACTCAAGAGGCTAT | TAAGGGTAGCTTCCCAGCCT |
| CD3E_off21 | AGAGGAGAAACAAAGGGAAAAGGT | CCACATCCTAACCTCTGCTGT |
